# Operationalizing vulnerability and resilience interdependencies in drought risk management

**DOI:** 10.1007/s11625-026-01859-z

**Published:** 2026-06-04

**Authors:** Abdullah Konak, Nazmiye Balta-Ozkan, Elisabeth Shrimpton, Michael Jacobson, Daniel Winstead, Adelaide Cassia Nardocci, Tanaya Sarmah, Eduardo Mario Mendiondo

**Affiliations:** 1 Information Sciences and Technology, The Pennsylvania State University, Berks, Reading, USA; 2 Faculty of Engineering and Applied Sciences, Cranfield University, Cranfield, UK; 3 Department of Ecosystem Science and Management, The Pennsylvania State University, University Park, USA; 4 School of Public Health, University of São Paulo, São Paulo, Brazil; 5 Department of Architecture and Planning, Indian Institute of Technology Roorkee, Roorkee, India; 6 São Carlos School of Engineering, University of São Paulo, São Paulo, Brazil; 7 School of Civil Engineering, University of Birmingham, Birmingham, UK

**Keywords:** Drought risk management, Vulnerability, Resilience, Vulnerability–resilience interdependence, Indicator-based framework

## Abstract

Droughts, exacerbated by climate change, pose increasingly complex challenges for social-ecological systems worldwide. Traditional index-based methodologies capture vulnerability and resilience at an aggregate level but often overlook the nuanced ways in which these two constructs interact. To address this gap, this study introduces the Matrix-Based Drought Indicator System (MADIS) framework that operationalizes the interdependence between vulnerability and resilience at the indicator level. By distinguishing between coping, adaptive, and transformative capacities, the MADIS framework supports a more detailed assessment of how specific resilience indicators can mitigate different vulnerability factors. Two studies, online workshops with global drought experts and collaborative workshops focusing on small-scale farming communities in Pennsylvania, demonstrate how participatory methods can be used to identify and validate these interdependencies. Findings suggest that integrating indicator-level interdependencies leads to more refined risk categorizations, facilitates stakeholder consensus, and better informs targeted policy interventions. These case studies also reveal the drawbacks and implementation challenges of the MADIS framework. Ultimately, the MADIS framework contributes to a more dynamic view of resilience and social-technical-ecological systems analysis of climate change-related risk assessment. Future work will expand upon this approach by incorporating continuous temporal feedback, additional hazard indicators, and integration with social-technical-ecological systems analysis.

## Introduction

Climate change has already reduced food security and water availability due to rising temperatures, shifting precipitation patterns, loss of ice and snow, and more frequent and severe weather events such as droughts (IPCC 2023b). Droughts are complex natural disasters with various direct and indirect effects. Drought hazards can be caused by arid weather, a significant lack of water, increased atmospheric water demand due to high temperatures or strong winds, or a lack of snow (Reduction 2021). These conditions directly impact the agricultural sector and harm other parts of the economy. Drought also leads to broader social and economic harm that can extend beyond affected areas (Tsegai et al. 2023). For example, in 2021, severe drought in the western United States resulted in agricultural losses of over $ 8 billion and affected water supplies for millions of people. Similarly, the 2010–2011 drought in the Horn of Africa caused food shortages for over 12 million people, leading to widespread malnutrition and migration (FAO 2011). With the expected increases in the frequency, severity, and duration of droughts due to climate change in many regions worldwide (IPCC 2023b; UNDRR 2020), it is highly likely that these costs will increase.

Beyond agricultural and economic systems, due to population increases and immigration, broader social-ecological systems are also more exposed to droughts. While the literature on drought vulnerability and resilience is extensive (see the reviews by Blauhut (2020), Dardonville et al. (2021) and González Tánago et al. (2016)), their interdependence is poorly understood. The Sendai Framework defines resilience as “*the ability of a system*,* community or society exposed to hazards to resist*,* absorb*,* accommodate*,* adapt to*,* transform and recover from the effects of a hazard in a timely and efficient manner*,* including through the preservation and restoration of its essential basic structures and functions through risk management*.” (UNDDR 2009). On the other hand, vulnerability involves “*the conditions determined by physical*,* social*,* economic*,* and environmental factors or processes that increase the susceptibility of an individual*,* a community*,* assets*,* or systems to the impacts of hazards*” (UNDDR 2009). In these definitions, vulnerability and resilience are interdependent: high vulnerability means greater susceptibility to hazards, reducing the system’s ability to resist and recover (lower resilience). Conversely, enhancing resilience by improving adaptive capacity and resources can reduce vulnerability. However, these relationships have not yet been clearly operationalized.

To develop a nuanced understanding of vulnerability–resilience interdependence without oversimplification (Gallopín 2006), it is important to recognize that each operates at different stages of risk analysis and complements the other in an inverse relationship (Zakour and Gillespie 2013). Promoting resilience during disaster response and recovery reduces future vulnerability (Graveline and Germain 2022; Lei et al. 2014; Zakour and Swager 2018; Simpson et al. 2021), while pre-disaster conditions strongly influence post-disaster recovery (Zakour and Swager 2018). One way to frame these dynamics is through the economic, social, structural, institutional, and human capital resources available across disaster phases (Norris et al. 2008; Zakour and Gillespie 2013; Zakour and Swager 2018). Vulnerability describes pre-disaster conditions that can deteriorate or be mitigated, whereas resilience focuses on processes during and after a disaster to recover or exceed pre-disaster functioning. Accordingly, vulnerability analysts emphasize economic and structural factors, while resilience analysts highlight social and institutional processes essential for equitable recovery (Norris et al. 2008). Both vulnerability and resilience researchers commonly adopt a social-ecological systems perspective because of the interconnections between social and environmental factors (Béné et al. 2012). In the context of drought, technologies such as early warning systems play an important role in enhancing resilience and reducing vulnerability (Kloosterman et al. 2021; Sundstrom et al. 2023), thereby informing a social-technical-ecological systems approach. This shared focus emphasizes understanding feedback loops where ecological processes impact social systems and vice versa, and how technology plays a role in regulating this relationship. Recognizing this feedback loop and the role of technology is crucial for developing strategies to enhance resilience and capacity to adapt (Béné et al. 2012) while reducing vulnerability.

Drought risk analysts frequently rely on indicator-based risk assessment approaches, in which a set of indicators is used to measure vulnerability and resilience. Linkage is generally sought at the aggregated levels of vulnerability and resilience. However, to date, the operationalization of linkages between vulnerability and resilience at the indicator level to better understand system responses to hazards and inform policy decisions has been overlooked. In addition, while indicator-based frameworks exist for drought risk assessment, they often focus on biophysical indicators; a comprehensive social-technical-ecological systems approach is needed for more effective policy decisions. To address these gaps, we propose a new framework, the Matrix-Based Drought Indicator System (MADIS), to represent and analyze the linkages between vulnerability and resilience at the indicator level. This new approach enables a more detailed and robust understanding of how specific indicators of vulnerability and resilience interact, thereby enhancing the accuracy of risk analysis and improving guidance for policy decisions. Focusing on interactions among individual indicators can also help evaluate the efficacy of alternative interventions and select specific ones to achieve a particular outcome. Specifically, this study addressed the following research questions:

1. *How can the interdependence between vulnerability and resilience be represented in indicator-based frameworks for drought risk analysis?*
2. *How does the effectiveness of resilience and vulnerability indicators in assessing and mitigating drought risk vary across different resilience dimensions?*
3. *To what extent does considering the interdependencies between vulnerability and resilience indicators at different dimensions enhance the predictive accuracy of indicator-based drought risk models?*

The paper is organized as follows: In “Background and literature review”, we review indicator-based drought assessment methodologies and case studies and the different approaches to linking vulnerability and resilience in drought assessment studies. “The MADIS framework for modeling linkages between vulnerability and resilience at the indicator level” presents our proposed MADIS approach. In “Materials and methods”, we present the research procedures for data collection and the empirical results from two case studies that examine the MADIS framework. “Results” presents the results and demonstrates that the proposed framework yields a more nuanced risk assessment approach. “Discussions” concludes with a discussion of the implications of our findings and the benefits and limitations of the MADIS approach.

## Background and literature review

### Definitions: indicator-based risk assessment, hazard, vulnerability, exposure, resilience, and risk

Different concepts and definitions of drought risk assessment and its components have been adopted (Hagenlocher et al. 2019). The IPCC (2023a) defines risk as “the potential for adverse consequences for human or ecological systems,” and it is usually estimated by the product of hazard, exposure, and vulnerability (Ehalt Macedo et al. 2022). Hazard is the potential occurrence of a natural or human-induced physical event or trend that may cause loss of life, injury, or other health impacts, damage, and loss to property, infrastructure, livelihoods, service provision, ecosystems, and environmental resources (IPCC 2023a). Vulnerability has been conceptualized and integrated into the drought risk assessment in various ways, and which can be broadly categorized under “outcome-impact” or “contextual-factor” approaches (Vogt et al. 2018). The outcome-impact approach aims to quantify a system’s vulnerability using historical impacts. The contextual-factor approach defines vulnerability as a function of an intrinsic state of the system, which is represented by exogenous variables such as “Exposure,” “Sensitivity,” and “Adaptive/Coping Capacity,” as follows:

$$\mathrm{Vulnerability}=f (\text{Exposure, Sensitivity, Adaptive/Coping Capacity})$$

Exposure is defined as the presence of people, livelihoods, species or ecosystems, environmental functions, services, resources, infrastructure, or economic, social, or cultural assets in places and settings that could be adversely affected (UNDRR 2020). Sensitivity measures the susceptibility of an exposed element to a hazard factor. The combination of exposure and sensitivity can lead to potential impact (Fontaine and Steinemann 2009), which can be mitigated by adaptive/coping capacity factors. Since the contextual-factor approach treats vulnerability as independent of external hazards (Brooks 2003), the resulting vulnerability metric represents the potential impact on the system. Combining this vulnerability with the frequency and severity of hazards provides a relative risk measure that can be used to compare multiple systems and evaluate alternative drought management and adaptation plans and policies (i.e., Risk = *f*(Hazard, Vulnerability)).

In terms of methods, a variety of quantitative and mixed-method approaches are employed in drought risk assessment (Blauhut et al. 2015; Hagenlocher et al. 2019). Among these, the indicator-based approach, which integrates indicators for hazard, exposure, and vulnerability, is a particularly valuable tool for measuring and comparing drought risk, as well as risks associated with other natural hazards and climate-related threats (Prabnakorn et al. 2019; Villani et al. 2022). The indicator-based approach provides a systematic and standardized method for evaluating and comparing multiple systems. Other methods, such as social-ecological models for analyzing system dynamics (Martin et al. 2016) and qualitative methods with participatory approaches, have been used in several situations (Ayantunde et al. 2015).

The indicator-based approach to drought risk assessment involves several steps. The process starts by identifying the objectives and context in which the risk index is used. The next step is to determine the indicators that represent the main components of the risk equation, typically hazard, exposure, and vulnerability. Drought hazard indicators are typically used to describe the drought conditions (severity, location, duration, and timing). Svoboda and Fuchs (2016) provide an extensive list of these indicators. A comprehensive list of vulnerability indicators is provided by Hagenlocher et al. (2019), Meza et al. (2019), and Sass et al. (2024). Once these indicators are selected, each is quantified or scored using available data or expert judgment. These values are then normalized to ensure comparability across indicators. Next, these standardized scores are combined into a single index using a formula that applies specific weights to each component, reflecting their relative importance to the overall risk. The resulting index provides an overall risk score, enabling comparison across regions or systems to identify where drought risk is most severe. This score can guide decision-makers in prioritizing areas that require more focused mitigation or adaptation measures. This summarized, indicator-based approach transforms complex, multidimensional data into a comprehensible format for strategic planning and policy formulation.

### Current approaches to integrating hazards, vulnerability, and resilience

In this section, we briefly summarize the major theoretical frameworks for understanding how vulnerability and resilience interact and are integrated, and situate MADIS relative to them. We group these frameworks in three broad categories: (i) conceptual framing models, (ii) causal frameworks that connect upstream conditions to downstream risk and policy response, and (iii) computational interdependence models that represent relationships among indicators or system components.

Conceptual framing models define what vulnerability/resilience mean and how they relate. As briefly described in the previous section, resilience is included as an aspect of adaptive capacity in the IPCC definition of vulnerability. The MOVE framework (Birkmann et al. 2013) aims to better conceptualize the complex nature of vulnerability by considering factors such as exposure, susceptibility, and lack of resilience across various dimensions (physical, social, ecological, economic, cultural, and institutional). The framework also integrates adaptation into disaster risk management as a distinct component of coping mechanisms. In the MOVE framework, vulnerability and resilience are integrated by treating them as interconnected components that influence one another within a system. This requires assessing vulnerability (exposure, susceptibility, and lack of resilience) and resilience (adaptive capacity and coping mechanisms) across multiple dimensions, including physical, social, ecological, economic, cultural, and institutional. However, MOVE does not provide mechanisms for indicator-level interdependence, as these dimensions remain parallel aggregates.

While the IPCC and MOVE frameworks highlight vulnerability, the 3-D resilience framework (Béné et al. 2012) provides an analytical method for understanding and enhancing resilience in systems facing adverse changes and shocks. The 3-D resilience framework consists of three core components: (i) absorptive capacity, the ability to buffer impacts and persist through coping strategies; (ii) adaptive capacity, the ability to make incremental adjustments to moderate potential damages or seize new opportunities; (iii) transformative capacity, the ability to create fundamentally new systems when existing structures become untenable. According to the 3-D framework, these components lead to different outcomes: persistence, incremental adjustment, and transformational responses. The framework suggests that resilience emerges from the combination of all three capacities. The 3-D framework is justified by the need to broaden the concept of resilience beyond simply “bouncing back” to include adaptation and transformation. The 3-D framework recognizes that different intensities of shock or change may require diverse responses, ranging from resistance to incremental adjustments to complete system overhauls. The 3-D framework has gained interest in recent years for modeling resilience in areas such as food systems resilience (Boerema et al. 2021), drought (Panchi Robles 2019), and small farmers (UNCCD 2022). MADIS complements the 3-D framework by operationalizing indicator-level representation of vulnerability–resilience that produces new quantitative metrics.

While the IPCC, MOVE, and 3-D frameworks aim to provide a comprehensive approach to vulnerability and resilience assessment, causal frameworks connect upstream conditions to downstream risk and policy response. These causal frameworks are mainly dynamic, focusing on understanding and addressing the root causes and hazards that lead to disasters. According to the Pressure-Release (PAR) framework (Blaikie et al. 2014), natural disasters occur at the intersection of two forces: the processes that create vulnerability and the physical exposure to hazards. The PAR model defines the process leading to vulnerability in three progressive stages: root causes (e.g., poverty, inequality, environmental degradation), dynamic pressures (e.g., inadequate water management, population growth, agricultural practices), and unsafe conditions (e.g., reliance on rain-fed agriculture, lack of irrigation infrastructure, limited water access). Typically, capacity is emphasized in the stages of addressing dynamic pressures and unsafe conditions. González-Tánago et al. (2016) note that most drought-vulnerability studies rarely connect science and policy: only a third involve end users, and only a third clearly link results to drought strategies. One approach to strengthen that connection is to use the Driver-Pressure-State-Impact-Response (DPSIR) conceptual framework, which structures environmental problems as a causal chain linking Drivers to Pressures, changes in State, resulting Impacts, and societal Responses (Tsakiris 2015). Similar to PAR, DPSIR traces how upstream societal conditions translate into downstream risks, but it emphasizes different parts of that chain to better understand and respond to people’s vulnerability to disasters. These frameworks are strong for diagnosis and communication, but they typically fall short of formalizing cross-indicator mitigation pathways.

Computational interdependence models represent relationships more explicitly, though with different logics. System dynamics approaches (Dougill et al. 2010; Lei et al. 2014) view vulnerability and resilience as interconnected components of a dynamic system, in which changes in one can influence the other. In such system dynamic models, the relationship between vulnerability and resilience indicators can be represented as a causal loop diagram. Understanding and managing feedback loops enables more proactive, adaptive responses to drought (Holman et al. 2021). Recently, the theory of self-organizing systems has attracted attention in the context of system resilience. For example, the Adaptive Cycle framework (Holling 2001; Gunderson 2002) provides insights into system resilience and capacity for adaptation by modeling how systems continuously organize, collapse, and reorganize. This framework comprises continuously looped exploitation, conservation, release, and reorganization phases, measured across three dimensions: potential (resources and capacity for growth), connectedness (strength of internal relationships and control), and resilience (ability to absorb disturbances and reorganize while maintaining its functions). Hyde et al. (2024) expanded the Quantifying the Adaptive Cycle (QtAC) model to create a more standardized and generic method for executing network analysis through dynamic systems in a broader range of systems with steps: (i) estimating information network structures, (ii) entropy by conditioning transition probabilities, (iii) ascendency analysis on information networks.

Bayesian networks (BNs) are also used to identify relationships among resilience and hazard variables by encoding conditional dependencies in a directed acyclic graph and updating beliefs as evidence changes (Mihunov and Lam 2020). For example, BN can learn the structure and drought-resilience parameters from county-level indicators and then run scenario-based conditional-probability queries that link hazard intensity, damage, and recovery (Mihunov and Lam 2020). Merritt et al. (2016) also use a BN, but rely more on expert judgment and surveys, introducing aggregate “capital strength” nodes to reduce the complexity of conditional-probability tables under data constraints. MADIS shares a similar goal because it encodes directed linkages between indicators. However, MADIS centers link resilience capacities to specific vulnerability outcomes, yielding clear intervention targets that can directly inform policy prioritization. By contrast, BN studies often emphasize relationships among resilience indicators or broader hazard–impact–recovery dependencies, rather than an explicit mapping of resilience-to-vulnerability mitigation for policy development.

### Indicator-based drought risk assessment

Most models of vulnerability and resilience discussed in “Current approaches to integrating hazards, vulnerability, and resilience” incorporate indicators that extend far beyond water availability. While hydrological and meteorological features may help predict drought hazards, they do not necessarily capture vulnerability or resilience to their impacts. It is now well-established that vulnerability and resilience involve a complex interplay of social, governance, and economic drivers (Adger 2006; Pelling 2012). Consequently, studies in this field are highly varied, often focusing on specific disciplines or issues. For example, some studies limit the system of interest to specific land uses, such as crop management (Yan et al. 2022), particular regions (Zarei and Mahmoudi 2022), or different scales, ranging from households (Ghimire et al. 2010) to global systems across Water-Energy-Food (WEF) systems (Mouratiadou et al. 2016). These studies employ a range of qualitative and quantitative methodologies. Regardless of the scope or disciplinary focus, state-of-the-art methods in building an indicator-based drought risk assessment can be analyzed in three stages: (1) Identification of Theories and Models, (2) Model and Indicator Selection, (3) Integration and Operationalization of Indicators.

In terms of theories and models (Stage 1), an illustrative example is Castell and Schrenk’s (2020) study of self-organizing systems, which draws on panarchy and the Adaptive Cycle metaphor (Gunderson 2002) to ground the study. They use computational methods and information theory to close the gap between the description of a complex, dynamic system and the quantitative measurements needed to assess it. The ecosystems case study applies a range of indicators to describe the system. This model and the thinking behind it underpin the study.

In Stage 2, selecting suitable indicators must be transparent and justified, as both chosen and excluded indicators shape a study’s outcomes. For example, results in Water-Energy-Food (WEF) system optimization models vary depending on whether studies focus solely on limited social indicators like labor costs (Yue et al. 2022) or incorporate human behavior factors (Bai and Sarkis 2022; Shi et al. 2020). Both approaches are valid but reflect different system boundaries. Unclear indicator selection can lead to biased results, as seen in reviews that found disproportionate emphasis on physical over social or governance aspects (Avraam et al. 2021), posing risks of skewed system optimization claims (Shrimpton and Balta-Ozkan 2024). Indicators should be supported by accessible, reliable, frequently updated datasets—a particular issue for drought indicators (Batalini de Macedo et al. 2023; Sass et al. 2024).

The third area for analysis (Stage 3) is integrating and operationalizing indicators. Once selected, indicators are combined into an index, requiring decisions on weighting and aggregation methods. Approaches are typically quantitative, participatory, or mixed. The weighted average is standard in drought risk studies, but indicator weights are set using various methods: equal, statistical, computational, qualitative, or hybrid. Quantitative/statistical methods are either objective, using data-driven calculations like Variance-based (Kukunuri et al. 2022), Entropy-based (Wang et al. 2020), Grey Relational Method (Bai et al. 2021; Gu and Ma 2016; Zhou et al. 2021), Principal Component Analysis (Arabzadeh et al. 2016; Norouzi et al. 2012; Topçu et al. 2022), and Copula (Kao and Govindaraju 2010), or subjective, relying on expert input, such as TOPSIS (Roshan et al. 2018; Sahana and Mondal 2023; Sahana et al. 2021) and AHP (Zarei and Mahmoudi 2022).

Subjective methods employ participatory approaches that leverage expert knowledge for indicator ranking, serving as alternatives or complements to other methods. These approaches help address data gaps, especially as climate change makes historical datasets less reliable for future predictions. By integrating diverse quantitative and qualitative data, participatory methods promote comprehensive and innovative thinking amid uncertainty (McPhearson et al. 2021). Common methodologies include AHP (Zarei and Mahmoudi 2022), Delphi (Crispim et al. 2022), Fuzzy Cognitive Mapping (Masinde et al. 2018), and other community-based participatory methods (Ghimire et al. 2010).

The choice of aggregation methods depends on several factors, such as the number and availability of experts, the number of indicators to be analyzed, available time and cost, and the researcher’s preferences. A core question in this context is how ‘expert’ is defined. In expert selection, decisions about whose views are considered and valued lead to questions of participatory justice and recognition (Schlosberg 2007). There are growing calls to expand expert knowledge by combining scientific and indigenous perspectives, especially in drought indicator research. This transdisciplinary approach helps tackle complex Anthropocene challenges like drought by appreciating diverse viewpoints. MADIS does not replace these weighting procedures defined about, rather, it introduces an additional structural layer that represents how resilience indicators mitigate specific vulnerability drivers.

## The MADIS framework for modeling linkages between vulnerability and resilience at the indicator level

In this section, we present an overview of our general strategy for modeling the linkages between vulnerability and resilience indicators to improve the guidance of drought risk management policy decisions. Hence, we will refer to this approach as the Matrix-Based Drought Indicator System (MADIS) framework in the forthcoming sections. This general strategy can be applied to specific scenarios by modifying how indicators are operationalized and variables are computed and connected. The overall objective is to compare *K* systems, *k*= {1,…,*K*}, based on a set of vulnerability and resilience indicators, denoted by sets *V* and *R*, respectively. A system may represent cities, regions, counties, periods, etc. The selection of *R* and *V* highly depends on the context and objectives. Let $\:{v}_{jk}$ and $\:{\rho\:}_{ik}$ be the values of vulnerability indicator *j* and resilience indicator *i* for system *k*, respectively. For the sake of simplicity, we assume that $\:{\nu\:}_{jk}$ and $\:{\rho\:}_{ik}$ are normalized in [0,1] using a min-max approach, where 0 represents the lowest levels of vulnerability and resilience, and 1 represents the highest levels. The overall resilience of system *k* is typically expressed as a weighted average of the indicators in *R* as follows:

$$\:{P}_{k}^{}=\frac{1}{\left|R\right|}\sum\limits_{i\in\:R}^{}{{w}_{i}\rho\:}_{ik}$$

where $\:{w}_{i}$ is the weight of indicator *i*, representing its importance or relevance in the overall resilience, and |*R*| is the cardinality of set *R.* Similarly, the overall vulnerability of system *k* is expressed as a weighted average of the vulnerability indicators in *V* as follows:

$$\:{V}_{k}^{}=\frac{1}{\left|V\right|}\sum\limits_{j\in\:V}^{}{{w}_{j}v}_{ik}$$

In earlier approaches to resilience, the focus has predominantly been on coping capacity or adaptive capacity. Building on the 3-D resilience framework (Béné et al. 2012), the MADIS framework expands on this traditional perspective by incorporating three distinct dimensions of resilience: coping capacity, adaptive capacity, and transformative capacity. Furthermore, in our framework, a resilience indicator’s effectiveness in mitigating potential harms can vary significantly depending on the type of capacity being considered. For example, a resilience indicator that is highly effective in enhancing coping capacity may not necessarily contribute equally to adaptive or transformative capacity. Similarly, an indicator that supports transformative changes may have a limited impact on immediate coping mechanisms. By distinguishing between these three capacities, we can better capture the multifaceted nature of resilience. Table 1 distinguishes these capacity types by their functions, time horizons & change, change mechanisms, and the vulnerability contexts they are intended to address, providing a clear interpretive guide for MADIS’s capacity-specific indicator linkages.

Let $\:{\beta\:}_{i}^{s}\:$represent the effectiveness of resilience indicator *i* under resilience dimension *s*, where *s*∈{1:coping, 2:adaptive, 3:transformative}, on a continuum from 0 to 1 such that 0 indicates no effectiveness and 1 indicates maximum effectiveness. Then, we can calculate the resilience of system *k* under dimension *s* as follows:

$$\:{P}_{k}^{s}=\frac{1}{\left|R\right|}\sum\limits_{i\in\:R}^{}{{\beta\:}_{i}^{s}{w}_{i}\rho\:}_{ik}$$

It should be noted that a resilience indicator *i* be effective for multiple capacity types (i.e., $\:{\beta\:}_{i}^{s}>0\:\exists\:\:s$).

At the next level, we also account for the vulnerability context within which resilience is assessed. For instance, a resilience indicator that is highly effective in enhancing the coping capacity of dealing with potential harm due to a vulnerability may be less effective in terms of adaptive capacity. We define matrix $\:{\boldsymbol{\beta\:}}^{\boldsymbol{s}}=\left\{{\beta\:}_{ij}^{s}\right\}$, where $\:{\beta\:}_{ij}^{s}\in\:\left[\mathrm{0,1}\right]$ represents the effectiveness of resilience indicator *i* in mitigating the potential harm from vulnerability indicator *j* under capacity type *s*. If $\:{\beta\:}_{ij}^{s}=0$, then resilience indicator *i* does not affect vulnerability indicator *j*, and if $\:{\beta\:}_{ij}^{s}=1$, then, resilience indicator *i* is highly effective in mitigating the harm caused by vulnerability indicator *j*. Matrix $\:{\boldsymbol{\beta\:}}^{\boldsymbol{s}}$ encodes cross-construct relationships from resilience to vulnerability, rather than a generic within-criteria influence network. This structure allows us to compute context-dependent resilience and adjusted vulnerability, where vulnerability is evaluated net of resilience’s mitigating effectiveness across capacities. Importantly, the same resilience indicator can be effective for one type of capacity but weak for another, and MADIS preserves this heterogeneity by using separate resilience dimensions rather than aggregating them into a single score. In the following section, we provide how matrix $\:{\boldsymbol{\beta\:}}^{\boldsymbol{s}}$ can be constructed through a participatory approach using different data collection methods.

In addition to leading a more granular risk assessment, the information encoded in $\:{\boldsymbol{\beta\:}}^{\boldsymbol{s}}$ has significant practical and policy implications. First, constructing $\:{\boldsymbol{\beta\:}}^{\boldsymbol{s}}$ involves a participatory approach, where different stakeholders provide input on the effectiveness of resilience measures and the impact of vulnerabilities. This not only ensures that $\:{\boldsymbol{\beta\:}}^{\boldsymbol{s}}$ reflects practical and contextual knowledge from various experts, policymakers, and community representatives but also enhances the systemic understanding of drought risk for those involved. Our experiences with two case studies demonstrated that the participatory approach to constructing $\:{\boldsymbol{\beta\:}}^{\boldsymbol{s}}$ allowed stakeholders and decision-makers, who usually work in isolation, to gain a more comprehensive perspective on drought risk management. Second, our approach also aims to tailor resilience strategies to specific vulnerabilities and capacities. Information coded in $\:{\boldsymbol{\beta\:}}^{\boldsymbol{s}}$ can better guide policymakers in allocating resources for capacity building and planning interventions to mitigate vulnerabilities, particularly those that cannot be directly addressed. As a result, an enhanced understanding of the linkages between resilience and vulnerability can lead to more effective and context-sensitive policy interventions, identifying which resilience levers most directly reduce which vulnerabilities and under what capacity while remaining compatible with indicator-based assessment practice. These operational elements are a contribution of MADIS to earlier risk assessment frameworks, such as MOVE or the 3D frameworks.

Once $\:{\boldsymbol{\beta\:}}^{\boldsymbol{s}}$ is constructed, we define a resilience efficiency metric in the context of vulnerability *j* and capacity type *s* for system *k* as follows.

$$\:{P}_{jk}^{s}=\frac{1}{\left|R\right|}\sum\limits_{i\in\:R}^{}{{\beta\:}_{ij}^{s}{w}_{i}\rho\:}_{ik}$$

The above formula for $\:{P}_{jk}^{s}\:$quantifies the overall effectiveness of resilience indicators in the context of vulnerability *j*. Hence, we define the context-dependent resilience of system *k* as follows:

$$\:{P^{\prime\:}}_{k}^{s}=\frac{1}{\left|V\right|}\sum\limits_{j\in\:V}^{}{P}_{jk}^{s}$$

The main difference between overall resilience metrics $\:{P}_{k}^{s}$ and $\:{P^{\prime\:}}_{k}^{s}\:$is that the latter considers the set of vulnerability indicators under consideration or used to define the system’s vulnerability. This approach leads to a more precise evaluation of resilience and enables policymakers to make informed decisions to enhance system resilience at different levels.

We can adjust the inherent vulnerability indicator $\:{v}_{jk}\:$by considering the mitigating effectiveness of the resilience indicators. This adjusted vulnerability for system *k* under capacity type *s* can be expressed as:

$$\:{v}_{jk}^{s}={v}_{jk}(1-{P}_{jk}^{s})$$

To obtain an overall measure of vulnerability for system *k* under capacity type *s*, we aggregate the adjusted vulnerabilities across all vulnerability indicators:

$$\:{V}_{k}^{s}=\frac{1}{\left|V\right|}\sum\limits_{j\in\:V}^{}{{w}_{j}v}_{jk}^{s}$$

**Table 1:** Conceptual summary of resilience capacities used in this study

Category	Coping capacity	Adaptive capacity	Transformative capacity
Core function (IPCC 2023b; UNDRR 2020)	Use available skills/resources to manage adverse conditions and maintain basic functioning	Adjust to potential damage/opportunities/consequences while maintaining the system’s basic identity and functioning	Create a fundamentally new system when existing ecological, economic, or social structures become untenable
Time horizon and change	Short-term: rapid response and stabilization.	Medium-term: incremental adjustment and learning. Emphasizes system improvements	Long-term: structural change and reorientation of goals, institutions, or policies. Emphasizes system redesign
Mechanisms (Béné et al. 2012)	Preparedness, early warning, emergency measures, buffering, redundancy, short-run continuity planning, access to immediate relief and services	Diversification, experimentation, learning-by-doing, incremental technical/practice changes, risk-spreading strategies, adjustments in management routines	Institutional and policy reform, governance redesign, reconfiguration of resource rights and incentives, major infrastructure or livelihood transitions
Vulnerability context	Acute shocks where timely response can prevent cascading impacts	Recurring or evolving stresses requiring broader integration across social–economic–environmental conditions	Systemic vulnerabilities (e.g., inequities, governance failures) where incremental adjustments are insufficient

## Materials and methods

In this section, we introduce the research procedures for studying and validating the MADIS framework described in The MADIS framework for modeling linkages between vulnerability and resilience at the indicator level.

### Procedures

**Fig. 1:**
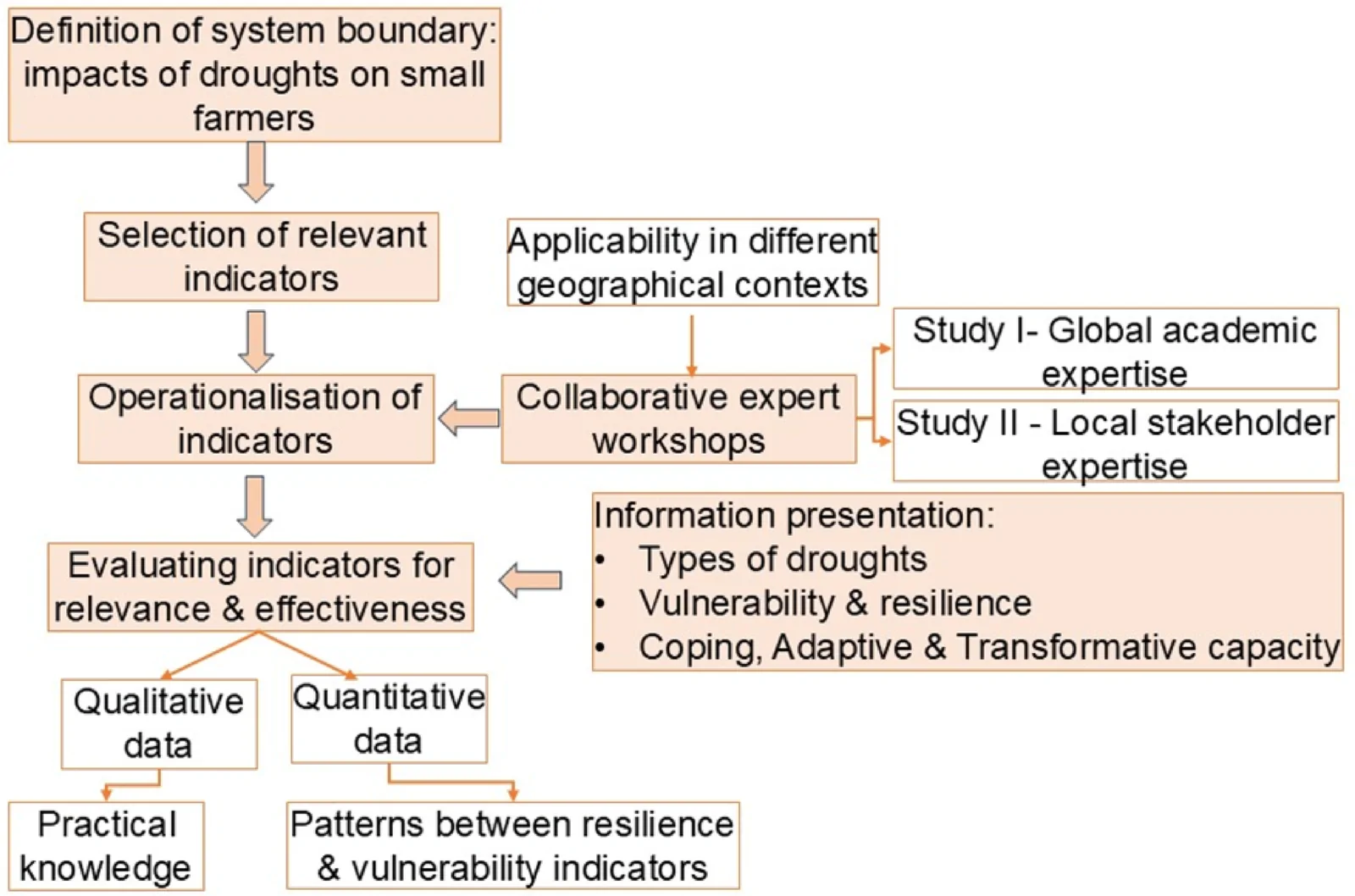
Research methodology

We applied a mixed research methodology by collecting qualitative and quantitative data in two online collaborative workshops and one face-to-face collaborative workshop to answer the research questions and study the validity of the MADIS framework and its suitability for real-world applications (Fig. 1). In order to make the framework applicable in different geographic contexts, these collaborative workshops involved global experts from academia (Study I) and local policymakers (government and local organizations, Study II). The recruitment process of the participants is discussed in the respective sections of the studies. In both studies, the participants provided quantitative input by evaluating indicators, categorizing them based on their relevance and effectiveness, and quantitative insights by discussing their implications within a real-world context. The collected quantitative data about the indicators and their relationships were analyzed to identify common patterns in the relationships between resilience and vulnerability indicators. The recorded text of these discussions was analyzed to capture insights grounded in practical knowledge, ensuring the framework’s relevance and applicability to diverse stakeholders. While Study I took place online, Study II took place in a face-to-face meeting, enabling local experts to evaluate the extent to which the drought resilience indicators applied to their region. Similarly, in the online workshops, participants from different countries shared insights relevant to small farmers in their countries. Both workshops began with an introductory presentation covering the core concepts of drought vulnerability and resilience, emphasizing key terms such as hazard, vulnerability, exposure, impact, and resilience. Additionally, the distinctions between coping, adaptive, and transformative capacities were clearly outlined. Participants were informed that the workshop would focus on small-scale farmers and the impact of drought on their livelihoods.

### Indicator selection process

In scoping these studies, we focused on small farmers given their critical role in global food production and heightened vulnerability to climate change. Small farms, often family-operated, produce around 80% of the world’s food (Lowder et al. 2021). However, these farms are more susceptible to climate-related hazards, particularly droughts, because they are primarily located in tropical regions and face socioeconomic and policy constraints that limit their ability to adapt (IPCC 2023a). Additionally, current frameworks for assessing small farms’ resilience can benefit from more theoretical approaches (Dixon and Stringer 2015). First, we conducted a comprehensive literature review focusing on drought vulnerability and resilience indicators relevant to small farms. We identified 136 indicators for drought vulnerability and resilience assessments across the dimensions of the Sendai Framework. We classified these indicator types (hazard, vulnerability, and resilience indicator) and categories (agricultural, government & policy, infrastructure & technology, social, socioeconomic, and water/stream). The research team members individually evaluated each indicator for its applicability to small farms and provided justifications for their decisions to include or exclude it from the final list. Through consensus-building meetings, 33 indicators were identified to be included in the study (for further details, please see (Batalini de Macedo et al. 2023; Sass et al. 2024). After consultation with outside experts, three other indicators were included. Tables A and B in the Appendix list the 17 resilience and 19 vulnerability indicators used in this study. The tables also provide how the indicators were calculated for Study II.

### Study I: Global online collaborative workshops

**Fig. 2:**
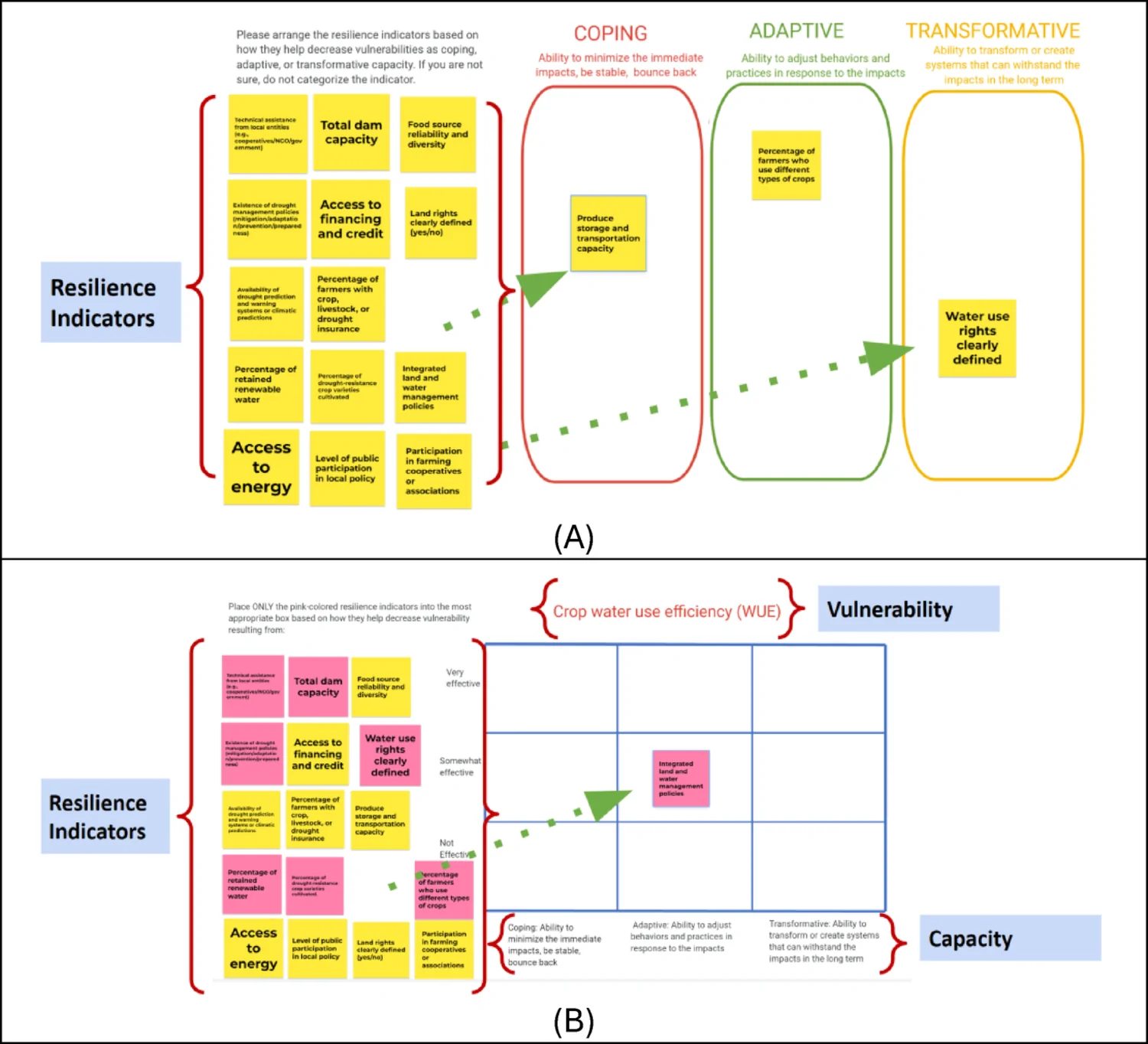
The layout of the interactive Jamboards used in the online workshop: **A** for categorizing the resilience indicators and **B** for defining the relationship between the resilience and vulnerability indicators

Two expert workshops (*N* = 6, *N* = 8) followed a semi-structured collaborative format with participation from different countries. They run between May and June 2023, each lasting approximately 1.5 h. Following the information presentation, the participants engaged in two group activities centered around drought vulnerability and resilience indicators. Discussions and participant engagement were facilitated using interactive Jam Boards (see Fig. 2).

The participants formed six groups of two or three. Each group was assigned to an independent Zoom room and a Google Jamboard, led by a moderator and a note-taker. In the first activity, using a consensus-building approach, the groups categorized 17 resilience indicators as coping, adaptive, or transformative capacity, based on their ability to mitigate vulnerabilities. The moderator’s role was to guide discussions, answer questions, and ensure the activity was completed on time. The primary goals of this activity were to familiarize participants with the indicators and to develop a shared understanding of the concepts of coping, adaptive, and transformative capacity before the second activity. Participants were only allowed to categorize each indicator into one category, not multiple. After reaching a consensus, the groups moved the resilience indicators to the correct groups on the Jamboard (Fig. 2A).

In the second activity, participants examined the linkages between vulnerability and resilience indicators. A total of 18 vulnerability indicators were chosen for this exercise. Comparing 17 resilience indicators with 18 vulnerability indicators was mentally demanding. To reduce participants’ cognitive load, the research team designed a partial factorial experiment before the workshop, using a subset of indicator combinations to capture key interactions and dependencies. Each group was assigned 5 to 6 vulnerability indicators and evaluated the relevant resilience indicators. This design made the evaluation manageable and enabled participants to focus on a limited set of pairwise comparisons without losing the broad insights needed for a thorough analysis. As shown in Fig. 2B, participants placed these resilience indicators in a 3 × 3 matrix, with the *x*-axis representing coping, adaptive, and transformative capacities, and the *y*-axis indicating their role in reducing vulnerability (not effective, somewhat effective, and very effective). For this process, a Jamboard was created for each vulnerability indicator. Participants also had the option to categorize each resilience indicator into multiple cells within the 3 × 3 matrix.

In the final activity, participants gathered in the main Zoom room to discuss their respective categorizations and justifications. The workshop concluded with an open discussion of the most challenging aspect of the activity for each group. Participants also discussed the pros and cons of the framework. These discussions were recorded for further qualitative analysis. The research team transferred the collected data from the Jamboard into CSV files for quantitative analysis.

### Study II: Face-to-face drought resilience workshops at the local level

The second case study also aimed to demonstrate the types of analysis that can be performed at the local level. It took place in Pennsylvania, USA, and involved (*N* = 18) participants from academia, state and local governments, and regional agricultural extensions and organizations in a face-to-face collaborative workshop format of approximately five hours. Before the workshop, the research team also collected data and computed a set of indicators for each of the 67 counties in Pennsylvania. During this collaborative workshop, participants also discussed the relevance of these vulnerability and resilience indicators in the context of Pennsylvania and identified the most appropriate data sources to evaluate them. The appendix provides the definitions, data sources, and the approach used to calculate those indicators.

Following the information presentation, in the first activity, the participants individually rated the relevance of the resilience indicators in the context of Pennsylvania on a 5-point Likert Scale from Not All (1) to Very Much (5). They categorized them as coping, adaptive, and transformative capacities, like in the online workshops. The second activity followed a similar process, in which participants evaluated and categorized the vulnerability indicators by their short-, medium-, and long-term impacts. Participants were provided with a conceptual definition of these impacts. We defined the short-term impacts of vulnerability as near-immediate effects occurring a few months after the onset of a drought. These are often the most visible and urgent impacts that require immediate action. Medium-term effects were defined as those that generally unfold over several months to a few years, representing the evolving consequences of prolonged drought conditions. These impacts often require adaptive strategies and more substantial interventions. Long-term (or structural impacts) were defined as those that persist for years to decades, potentially altering the fundamental characteristics of ecosystems, economies, and societies. Participants were allowed to categorize the indicators into multiple groups. In the final activity, the participants discussed the overall framework and linkages among the vulnerability and resilience indicators. The workshop concluded with an open discussion about the framework and drought management challenges.

## Results

### Results of study I

#### Study I: Analysis of quantitative data and findings

**Fig. 3:**
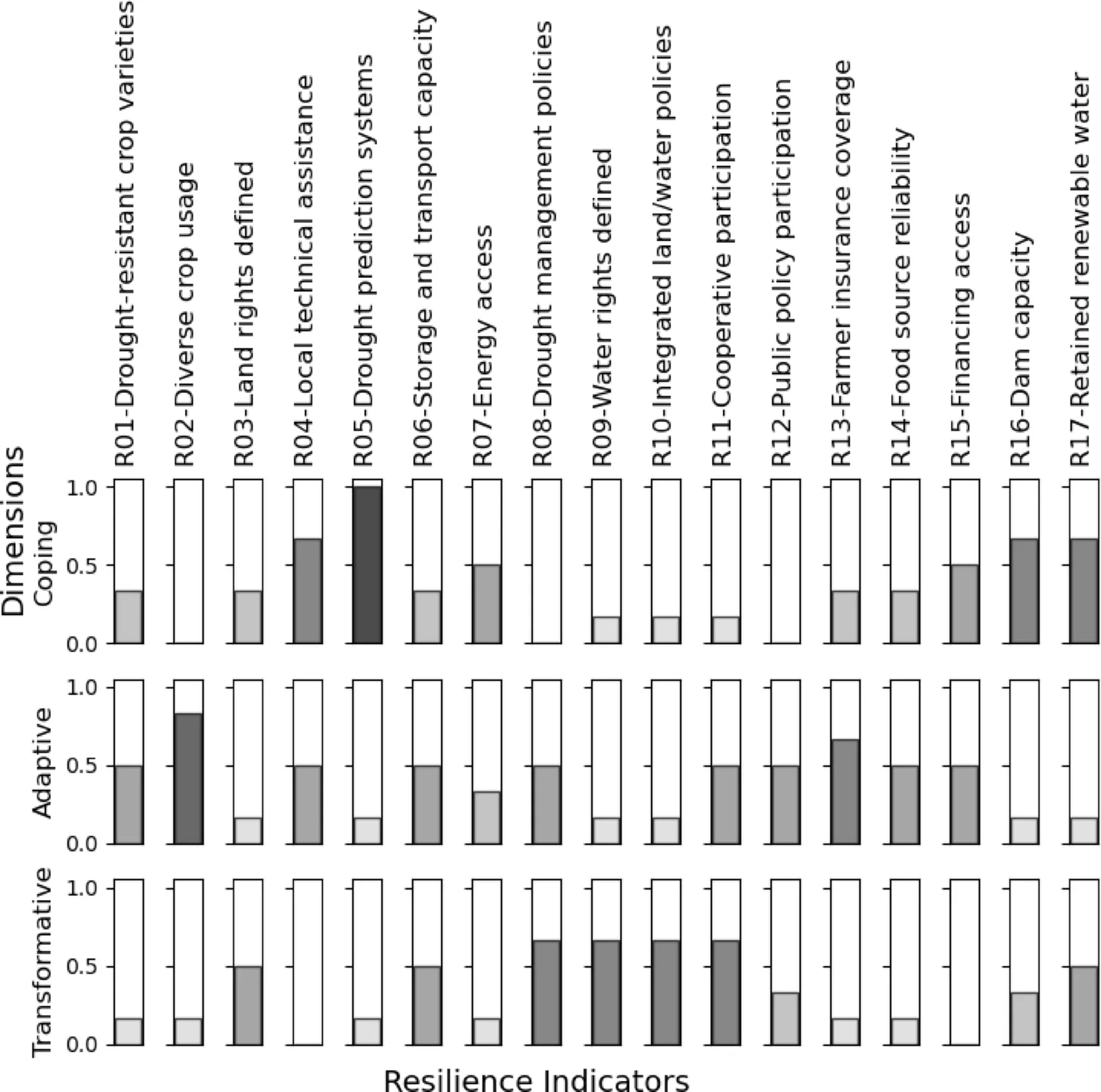
The average ratings of the resilience indicators across coping, adaptive, and transformative dimensions

First, we analyzed the classification of the resilience indicators by averaging the categorization of the groups across the three dimensions, coping, adaptive, and transformative capacity (i.e., calculating $\:{\beta\:}_{i}^{s}\:$in our framework). The results are given in Fig. 3. This provided insights into resilience and practical strategies for handling immediate shocks, enabling medium-term adjustments, and supporting long-term systemic transformations. Several patterns were observed in this respect. Indicators R05-Availability of drought prediction and warning systems (1.0), R04-Technical assistance from local entities (0.67), R16-Total dam capacity (0.67), and R17-Percentage of retained renewable water were identified as most effective at minimizing immediate impacts of drought in the coping dimensions. Indicators R16 and R17 are related to immediate water availability to prevent crop failures and livestock losses. In the adaptive capacity dimension, many indicators had average values of 0.5 or higher, while R02-Percentage of farmers who use different types of crops (0.83) and R13-Percentage of farmers with crops, livestock, or drought insurance (0.67) stand out. Indicators with a rating above 0.5 in the adaptive capacity dimension enhance farmers’ flexibility and ability to respond dynamically to drought challenges. These indicators also had various domains: agricultural, socioeconomic, infrastructure, and policy, and represented more proactive strategies. In the transformative capacity dimension, the highest rated indicators were in the policy domains, including R09-Water use rights clearly defined (0.67), R10-Integrated land and water management policies (0.67), and R08-Existence of drought management policies (0.67). This result implied the importance of effective governance in driving systemic changes to reduce future vulnerabilities.

Next, we calculated the effectiveness of the resilience indicators in mitigating the potential harm due to vulnerabilities under each capacity type based on the data collected in the second participatory activity (i.e., calculation of $\:\left\{{\beta\:}_{ij}^{s}\right\}$ in our framework). $\:{\beta\:}_{ij}^{s}$ values were derived from the Jamboard exercise in which workshop groups rated how effective each resilience indicator * i* would be in mitigating each vulnerability indicator $\:j\:$under a given capacity type * s* (coping, adaptive, transformative). Ratings were coded numerically “not effective”-0, “somewhat effective”-1, and “very effective”- 2. For each $\:\left(s,i,j\right)$ combination, we aggregated responses by taking the mean of the coded ratings across groups. Finally, the resulting averages were normalized to the [0,1] interval by dividing by 2, yielding $\:{\beta\:}_{ij}^{s}$ values interpretable as a standardized effectiveness score for resilience indicator * i* in reducing vulnerability indicator $\:j\:$ within capacity type * s*.

**Fig. 4:**
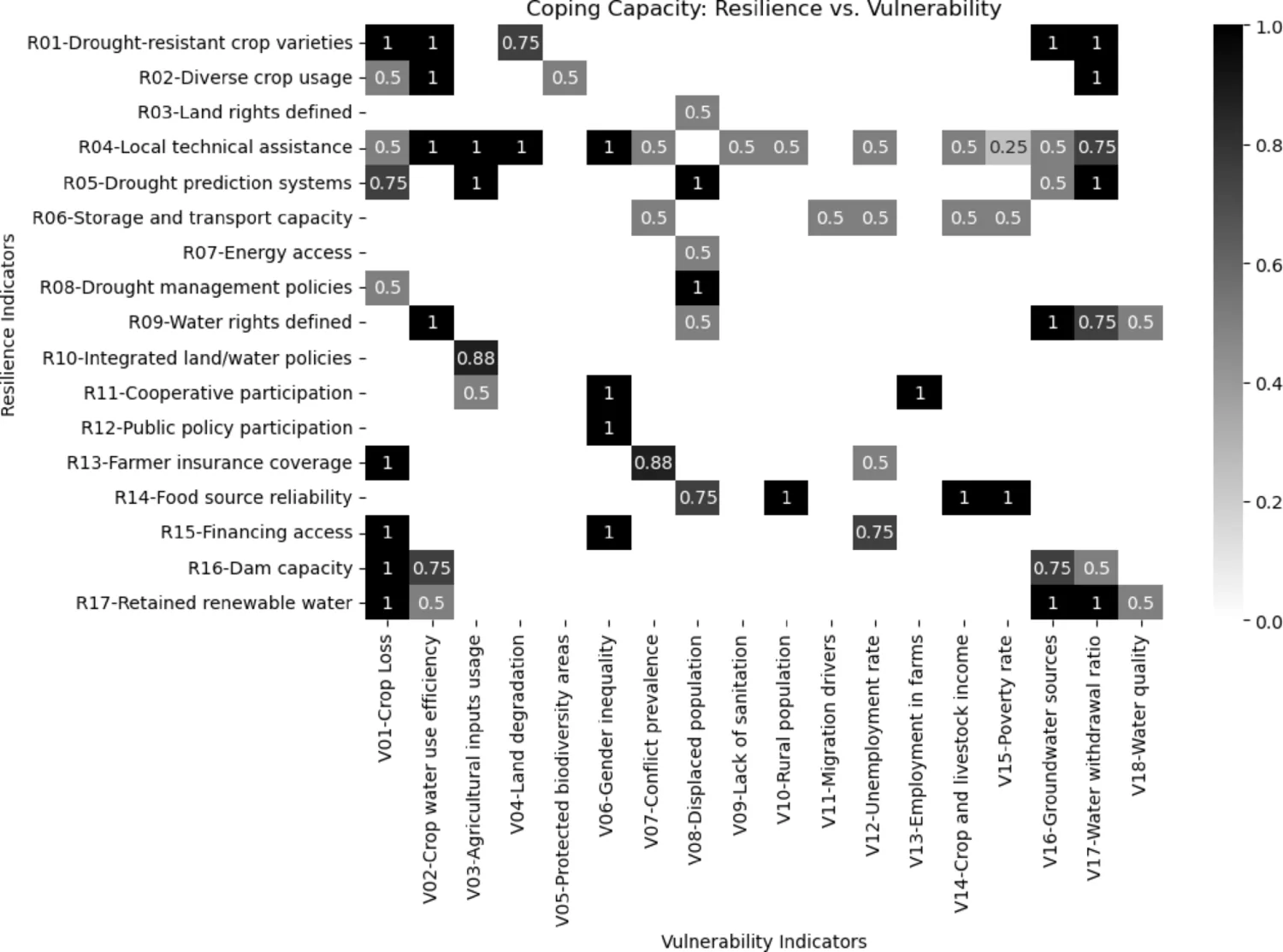
The average normalized ratings for the relationships between the resilience and vulnerability indicators at the coping capacity dimension

The patterns and relationships observed in the coping dimension (Fig. 4) showed that the effectiveness of the resilience indicators was unevenly distributed across the vulnerability indicators. Vulnerability indicators related to social and environmental systems (e.g., V06-Gender Inequality, V07-Conflict Prevalence, V09-Lack of Sanitation, V10-Rural Population, V13-Employment in Farms) were covered by a few resilience indicators. Some resilience indicators showed moderate-to-high effectiveness across multiple vulnerability indicators, such as R01-Drought-resistant Crop Varieties, R04-Local Technical Assistance, and R05-Drought Prediction Systems. Such indicators can be considered suitable for broad coping strategies. Policy-related resilience indicators such as R03-Land Rights Defined and R12-Public Policy Participation had a more focused relationship with only a single vulnerability indicator at the coping dimension. Water-related resilience indicators had particularly strong linkages with water-related vulnerabilities, indicating that water management is a critical focus in coping strategies.

**Fig. 5:**
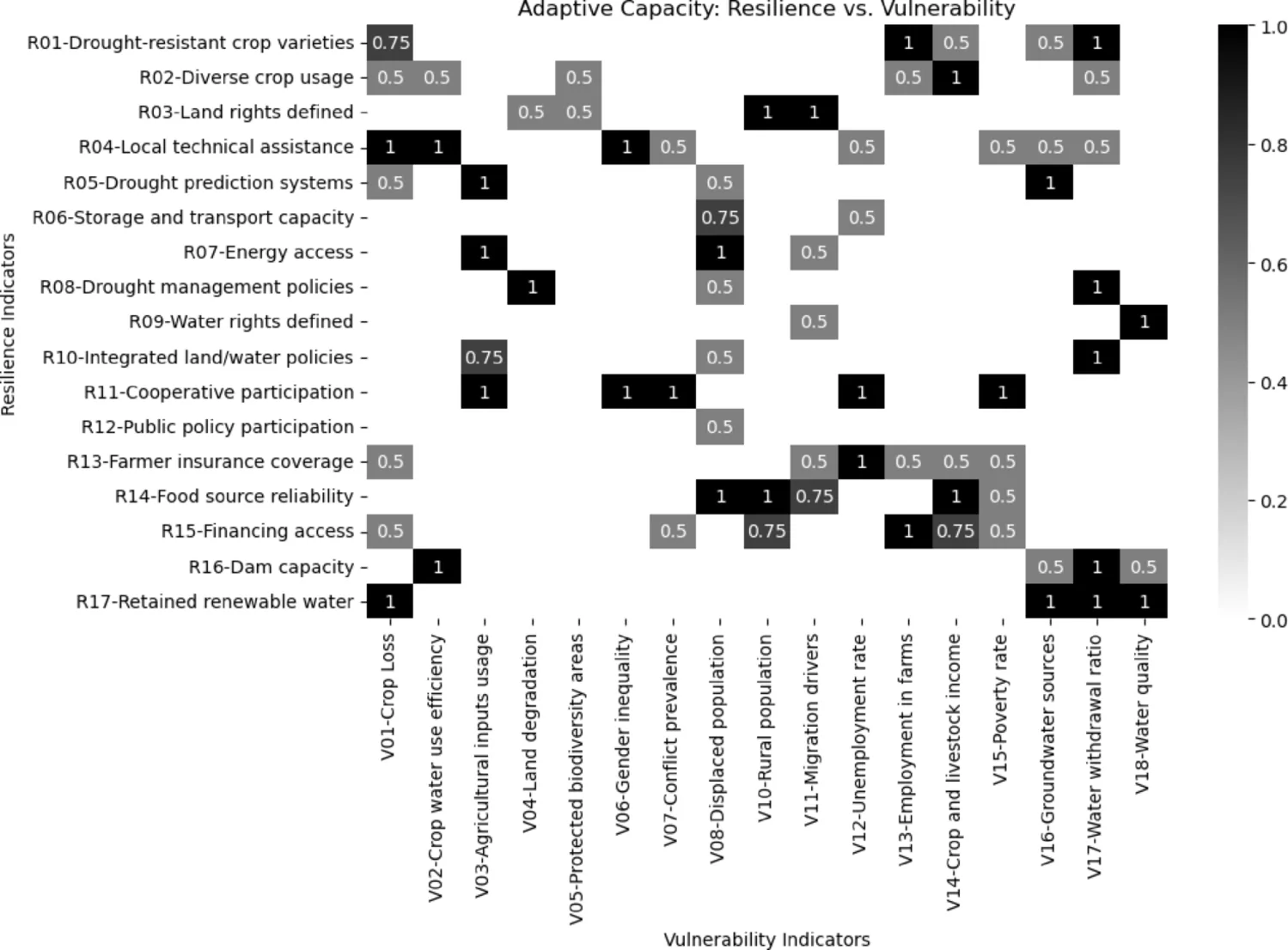
The average normalized ratings for the relationships between the resilience and vulnerability indicators at the adaptive capacity dimension

Figure 5 presents the calculated average normalized relationships at the adaptive capacity dimension. The adaptive capacity relationship matrix had similarities with the coping capacity relationship matrix, but a few new relationships emerged. Again, the water-related resilience indicators showed a strong relationship with the water-related vulnerabilities, underscoring the importance of water management in both coping and adaptive capacities. Indicators like R04-Local Technical Assistance were also related to many vulnerability indicators, and social and economic indicators such as R11-Cooperative Participation and R13-Farmer Insurance became more significant in the adaptive capacity dimension. This suggests that social cohesion and financial stability are crucial for adapting to drought. Similarly, a broader set of resilience indicators became associated with social and environmental vulnerability indicators, whereas this was not the case for the coping capacity dimension. As a result, the adaptive capacity relationship matrix was denser compared to the coping capacity matrix, suggesting that adaptive strategies require a broader and more integrated approach to address complex relationships between social, economic, and environmental vulnerabilities. On the other hand, the R05-Drought Prediction Systems indicator became more targeted at the adaptive capacity dimension.

**Fig. 6:**
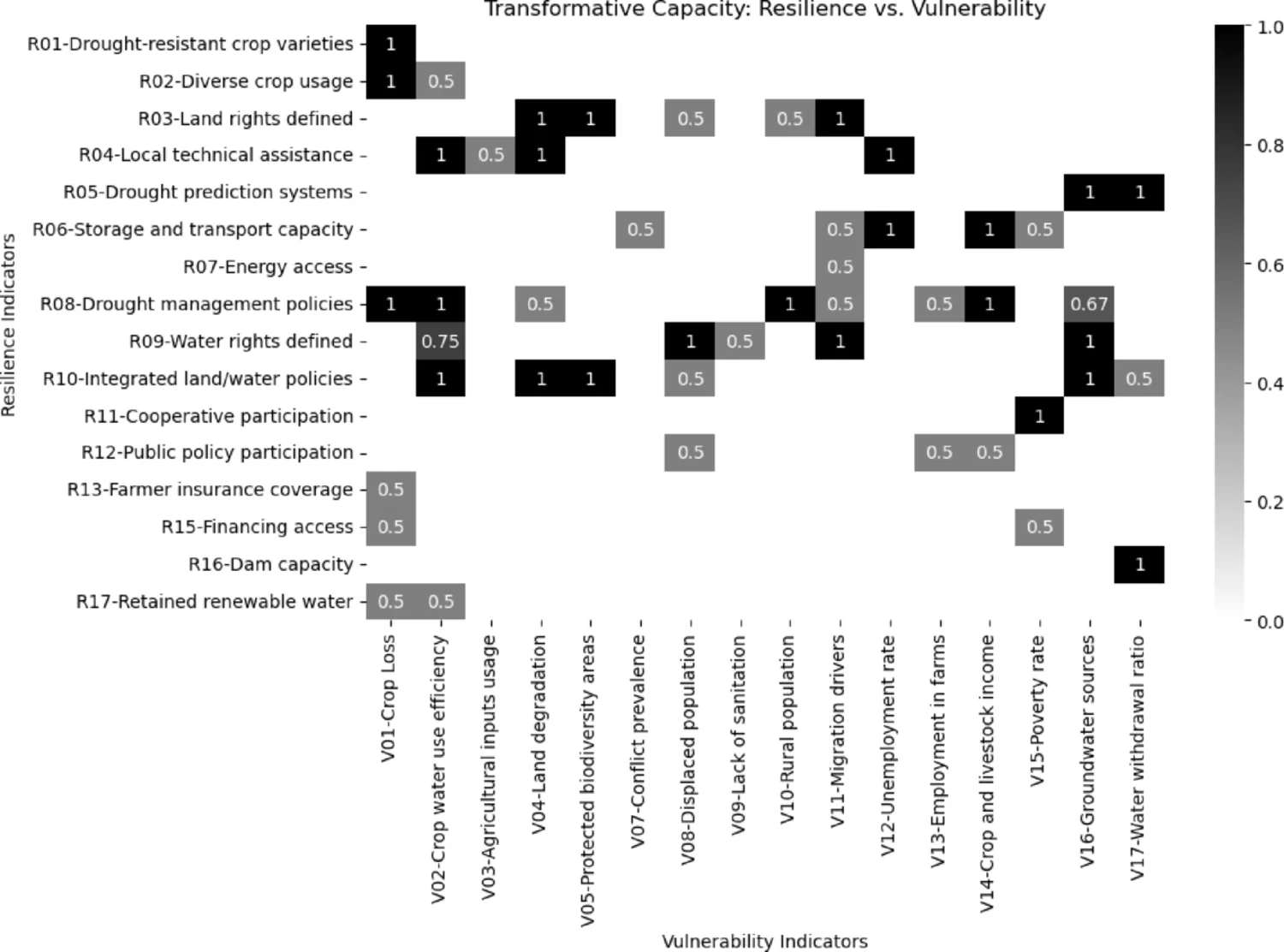
The average normalized ratings for the relationships between the resilience and vulnerability indicators at the transformative capacity dimension

Figure 6 presents the relationship matrix for the effectiveness of the resilience indicators in addressing vulnerability indicators at the transformative capacity level. Parallel to the results of the first activity, policy-related indicators such as R03-Land Rights Defined, R10-Integrated Land and Water Policies, and R08-Drought Management Policies became more prominent. These resilience indicators were strongly connected to social, socioeconomic, and environmental vulnerability indicators. The strong relationship between agricultural vulnerability and resilience was also observed in this dimension.

Overall, the results of these online collaborative workshops showed that the relationships among the resilience and vulnerability indicators are complex and depend on the context of the capacity dimension. The proposed framework can model these complex relationships across multiple dimensions, integrate context-specific insights, and provide tailored policy guidance. The workshops also demonstrated that it is possible to develop useful metrics to support policymakers in navigating the complex relationships among indicators. Such an analysis can help them identify gaps, prioritize interventions, and allocate resources effectively in line with specific policy objectives (e.g., whether to prioritize increasing coping or supporting transformative strategies). In addition, we observed improved stakeholder engagement through consensus-building during the workshops. This kind of engagement can lead to broader acceptance of planned resilience strategies and increase the likelihood of their successful implementation.

#### Study I: Analysis of qualitative data and findings about the strengths and challenges of the MADIS framework

At the end of the online workshops, we moderated discussion sessions to explore the potential strengths and weaknesses of the proposed approach. Participants were asked specific questions such as “What are the challenges in categorizing indicators and mapping the relationships?”, “What do you think about this framework?”, and “What are the pitfalls?“. The discussions were recorded and transcribed electronically. The transcripts were then analyzed using an inductive approach to identify significant emerging themes, which are given in Tables 2 and 3. Overall, the discussions highlighted the consensus on the importance of integrating resilience and vulnerability indicators to create a comprehensive framework for evaluating drought resilience. The participants noted the strength of the framework for its more nuanced approach, the requirement for interdisciplinary thinking, and the promotion of collaborative work (Table 2). The participants recognized and emphasized this objective and rationale of the framework. Nevertheless, they also highlighted the need for flexibility and adaptability in the framework, incorporating various indicators and adjusting to different contexts and data sources (Table 3). One of the unique challenges of the framework is the complexity of quantifying the relationship between resilience and vulnerability in particular regions and contexts, as these interactions cannot be easily generalized. However, defining the linkages between resilience and vulnerability can also lead to an unexpected benefit: the potential for the framework to promote interdisciplinary collaboration and encourage stakeholders to consider diverse perspectives in a comprehensive risk assessment. Some of the challenges pointed out by the participants were due to time constraints during the study (1.5 h). Clearly, the implementation of the framework requires multiple, longer workshops and should include sessions on selecting indicators most appropriate to the local context.

**Table 2:** Practical insights into the strengths of the MADIS framework

Theme	Explanation	Sample statements
Nuanced understanding of drought risk	The framework encouraged more detailed and nuanced discussions about resilience and vulnerability The participants agreed that mapping relationships between vulnerability and resilience indicators encouraged a more systemic understanding of how different factors interact in drought risk scenarios. This allowed participants to consider the complex interplay among environmental, social, economic, and policy factors	*“It did push a lot. And it is good for us to have that more nuanced discussion"*
Interdisciplinary Thinking	The participants noted that the proposed approach pushed them to consider multiple aspects of drought resilience. The proposed approach integrates biophysical indicators with policy and adaptation concepts, which required participants to think across disciplines Overall, the framework challenged participants to step outside their usual perspectives and consider how different factors interact in complex drought resilience scenarios	*“I come from the biophysical modeling side. So the challenge is*,* of course*,* I’ve never seen the other side of the story. Now we have shown me that part.”*
Collaborative Approach	The process of identifying relationships was considered highly beneficial due to its collaborative nature. The participants underscored the necessity of employing a multidisciplinary approach to conduct a comprehensive risk assessment They commended the proposed approach for its capacity to facilitate participatory methodologies and integrate diverse experiences and perspectives to analyze the relationship among the indicators	*“I feel like risk assessment doesn’t come often with collaborative methods. And this is very important to introduce new knowledge and to reduce uncertainty.”*

**Table 3:** Practical insights on the weakness of the MADIS framework

Theme	Explanation	Sample statements
Contextual and data availability challenges	The framework may not be universally applicable due to local differences in community and farmer conditions and opinions, policy and data availability Concerns for the lack of reliable or timely data in many countries limiting the framework’s effectiveness	*“We need to know where the context is*,* where the place is… I mean*,* policies are locally driven and understanding the physical*,* bio-physical system and then people who are residing in it. So every place is different.”* *“If you define clearly the indicators*,* it would be better to get precise information. But even if you [have] precise indicators*,* you will have the issue that many of our countries*,* I would say most of the countries in the world*,* do not have reliable data or timely data.”*
Indicator specificity vs. adaptability	Specific indicators might limit the framework’s adaptability to different local contexts	*“I feel like you want it to be adaptive to diverse systems. So*,* you know*,* other states and other management teams can apply to different systems. And maybe their applicability*,* if it is too specific to a certain group*,* maybe [its applicability will be] compromised?”*
Complexity in quantification and categorization	Difficulty in categorizing the indicators and quantifying relationships. Specific domain knowledge expertise leading to inconsistencies in the quantification of the relationships	*“…everything seems like coping for me because every time you’re going through a disaster*,* like everything should be immediate.”* *“We are talking about biophysical process*,* [and] then policies and adaptation*,* transformation. So it’s [kind of an] interdisciplinary thing. So*,* coming to terms with a common language to really see what we are seeing through different prisms… to bring ourselves up to speed was a challenge”.*

### Results of study II

#### Study II: Summary of the quantitative findings

**Fig. 7:**
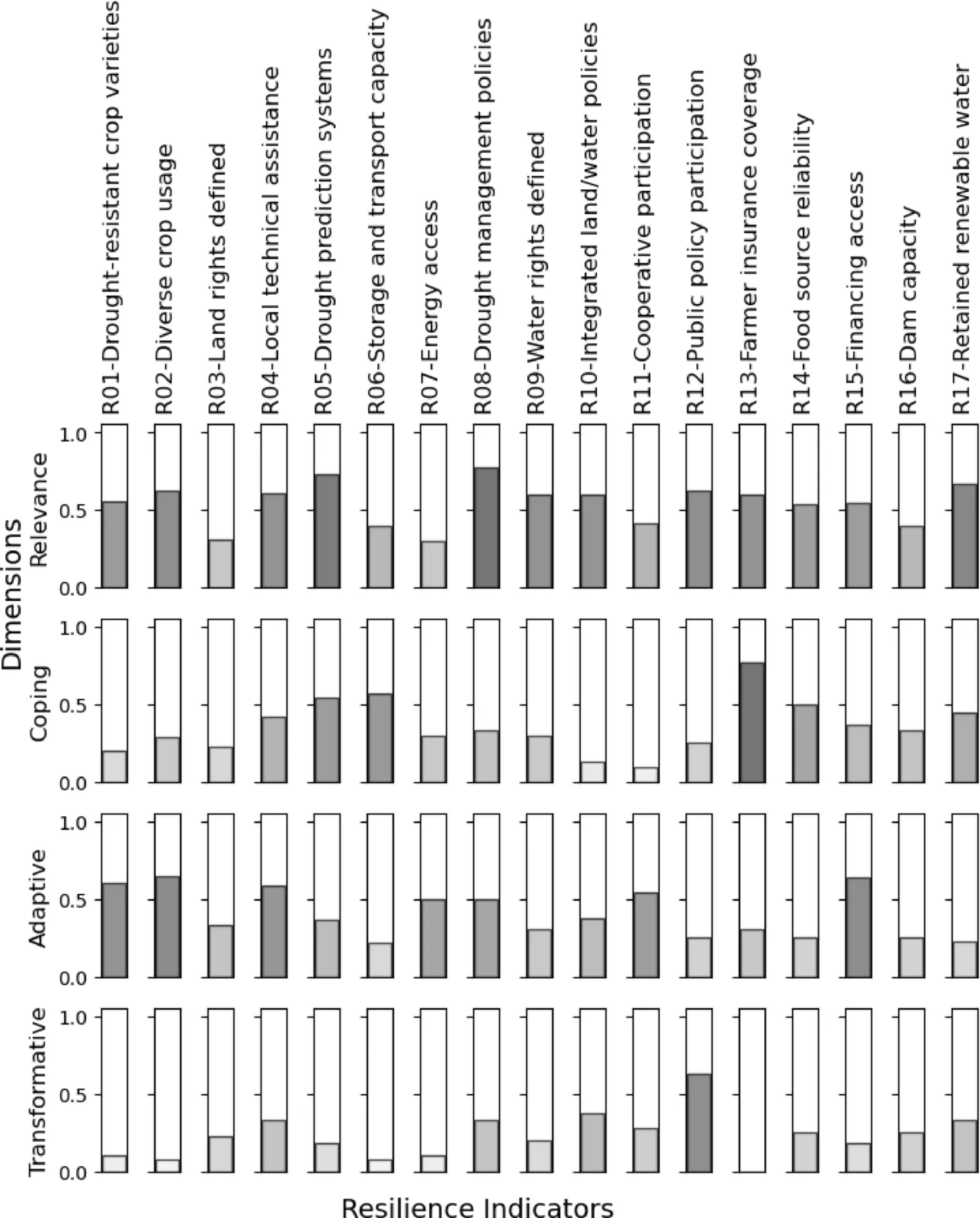
The normalized average of relevance, coping, adaptive, and transformative capacities of the resilience indicators

**Fig. 8:**
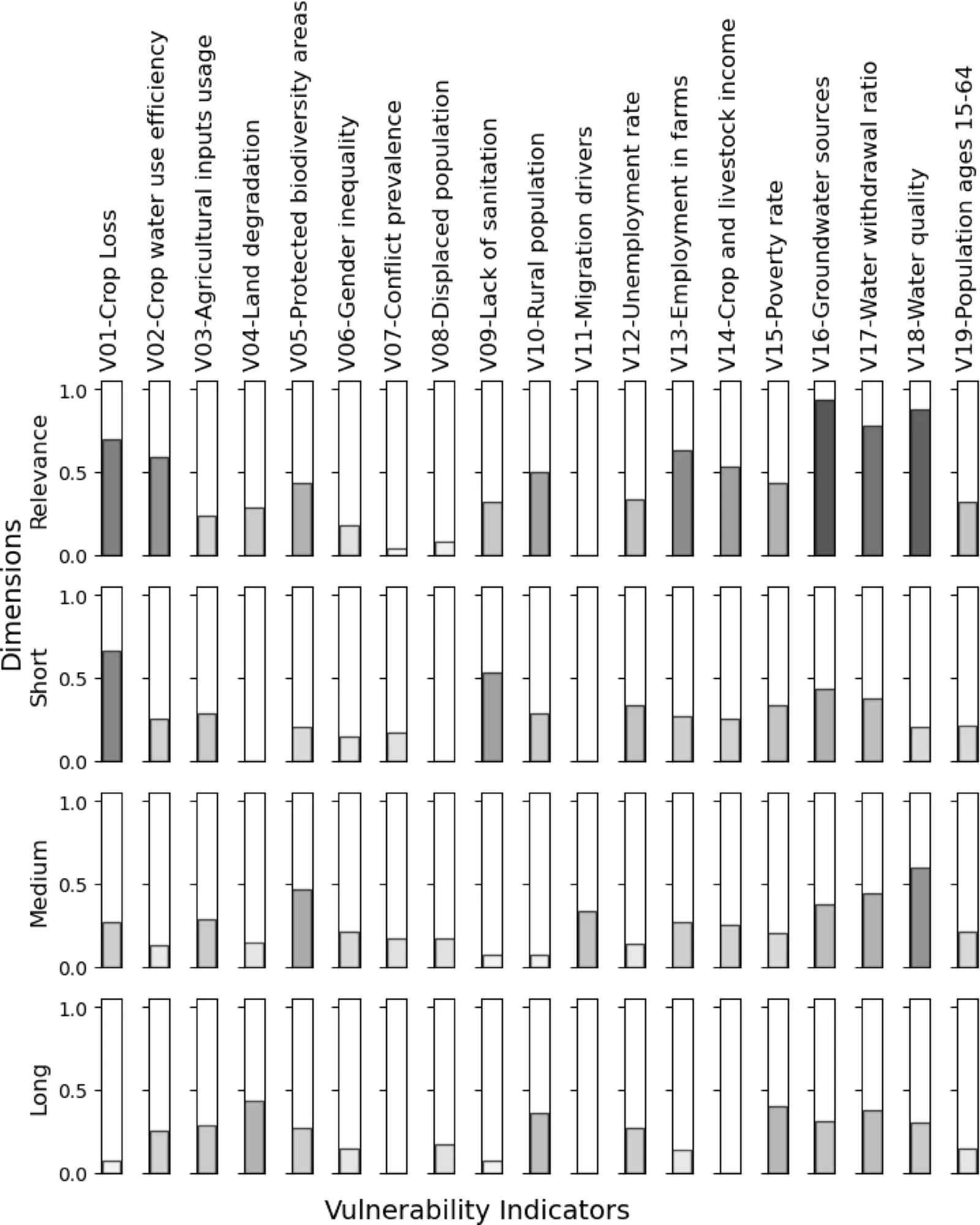
The normalized average of relevance, short, medium, and long-term impact vulnerability indicators

During the workshop activities, three types of quantitative data were collected and processed. First, the relevance scores for each indicator were determined using participants’ ratings on a 5-point Likert scale, ranging from “Not at All” (1) to “Very Much” (5). The $\:{w}_{i}$ values were calculated by averaging all participants’ relevance responses for each indicator, providing a measure of perceived importance in the Pennsylvania context. Second, categorization ratios were derived from how participants classified the indicators. For vulnerability indicators, these ratios represented the proportion of responses falling into short-term, medium-term, and long-term impact categories, denoted by $\:{\gamma\:}_{j}^{t}$ where *t*={short, medium, and long}. For resilience indicators, the ratios reflected the distribution across coping, adaptive, and transformative capacities (i.e., $\:{\beta\:}_{i}^{s}$ as defined above). Finally, these calculated weights and ratios were normalized to the range [0,1]. Figures 7 and 8 illustrate the normalized average values of the indicators. This data collection approach is an alternative to directly eliciting all pairwise resilience-vulnerability linkage scores, which was a cognitively heavy process as discussed in Table 3. From $\:{\gamma\:}_{j}^{t}$ and $\:{\beta\:}_{i}^{s}$, the linkage scores can be derived using factorization as a parsimonious surrogate approach, which reduces degrees of freedom while preserving interpretability and provides a feasible alternative if the required expertise time is limited.

We observed some similar patterns with Study I but also some subtle differences. In terms of coping capacity, R05-Drought Prediction Systems (0.55), water-related indicators (R16 - Dam Capacity (0.33) and R17-Retained Renewable Water (0.44), R13-Farmer Insurance Coverage (0.77)) were among the most critical indicators, similar to the online expert workshops in Study I. A subtle difference was that R06-Storage and Transport Capacity (0.57) was considered more a coping capacity than an adaptive capacity, and the role of insurance was more prominent. At the same time, the water-related indicators were relatively less emphasized in the coping dimension.

Indicators such as R01-Drought-resistant Crop Varieties (0.60), R02-Diverse Crop Usage (0.64), R04-Local technical assistance (0.58), and R15-Financing access (0.64) were identified as necessary in terms of facilitating adaptation to drought conditions. This relationship was also observed in Study I. In the context of adaptive capacity, multiple resilience indicators were considered to enhance farmers’ flexibility and their ability to respond dynamically to drought challenges. A difference was that policy-related indicators R08-Drought Management Policies (0.50) and infrastructure and technology indicators (R04-Local Technical Assistance (0.58) and R07-Energy Access (0.50)) were highlighted slightly more compared to Study I.

In the transformative capacity dimension, many indicators were found to be ineffective, excluding R12-Public Policy Participation (0.63). In this dimension, policy-related indicators had a higher importance score compared to other indicators but not to the level observed in Study I. In the global expert workshop, water-related indicators were crucial for coping, whereas Study II showed relatively lower significance for coping but higher significance for transformative capacity. Below, we provide the rationale for these subtle differences and analyze the relationships among the indicators.

Among the vulnerability indicators with high relevance scores, V01-Crop Loss and V16-Groundwater Level/Sources suggest that the negative effect of the drought would be immediately observable due to vulnerabilities in these indicators. V09-Lack of Improved Sanitation may also cause immediate effects, yet this indicator has medium relevance in the context of Pennsylvania. Indicators like V17-Water withdrawal ratio and V16-Groundwater Level/Sources had medium and long-term values as high as 0.937, suggesting that vulnerabilities related to these indicators can have medium to long-term impact and may become critical as drought persists. V15-Poverty rate, V10-Rural population, and V3-Agricultural Inputs usage have a medium relevance score; however, drought can result in lasting structural changes and can have a long-term impact due to the vulnerabilities represented by these indicators.

Next, we used the temporal relationships defined in the 3-D resilience model (Béné et al. 2012) between the indicators to identify $\:{\boldsymbol{\beta\:}}^{\boldsymbol{s}}$ matrix based on the notation that coping mechanisms address immediate vulnerabilities, while transformative resilience tackles long-term structural vulnerabilities. Adaptive capacity bridges the gap between short-term coping mechanisms and long-term transformative change by representing a system’s ability to adjust to changing conditions, mitigate potential damage, and address consequences. The calculation of $\:{\beta\:}^{s}$ is given as follows:

$$\begin{aligned}{\beta\:}_{ij}^{s}&={\beta\:}_{i}^{s}{\gamma\:}_{j}^{t}\:\:\forall\:\left(s,t\right)\in\:\{\left(Coping,Short\right),\\ & \quad \left(Adaptive,Medium\right),\left(Transformative,\:Long\right)\}\end{aligned}$$

**Fig. 9:**
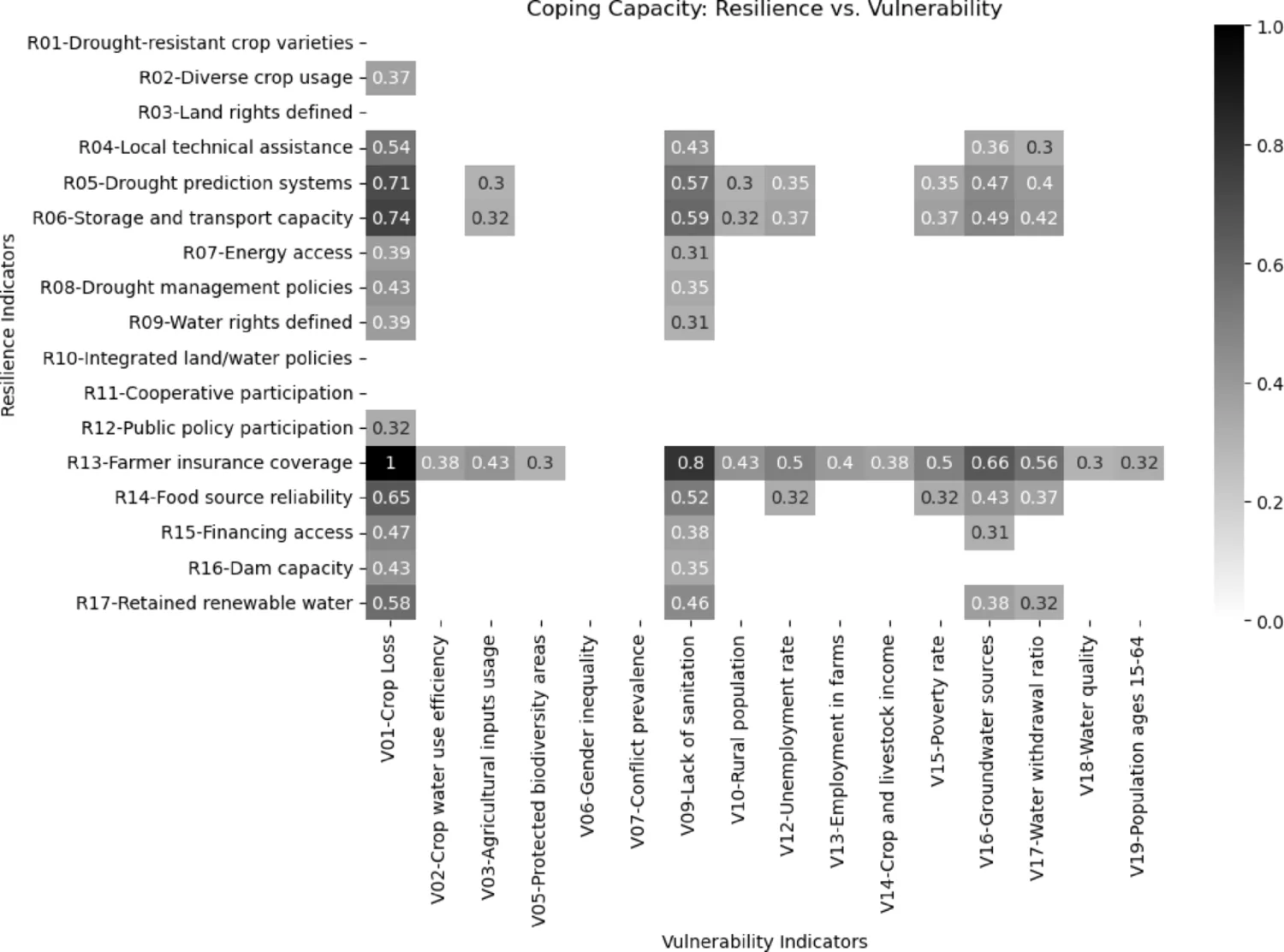
The average normalized ratings for the relationships between the resilience and vulnerability indicators at the coping capacity dimension (values < 0.3 were omitted for the clarity of the presentation)

**Fig. 10:**
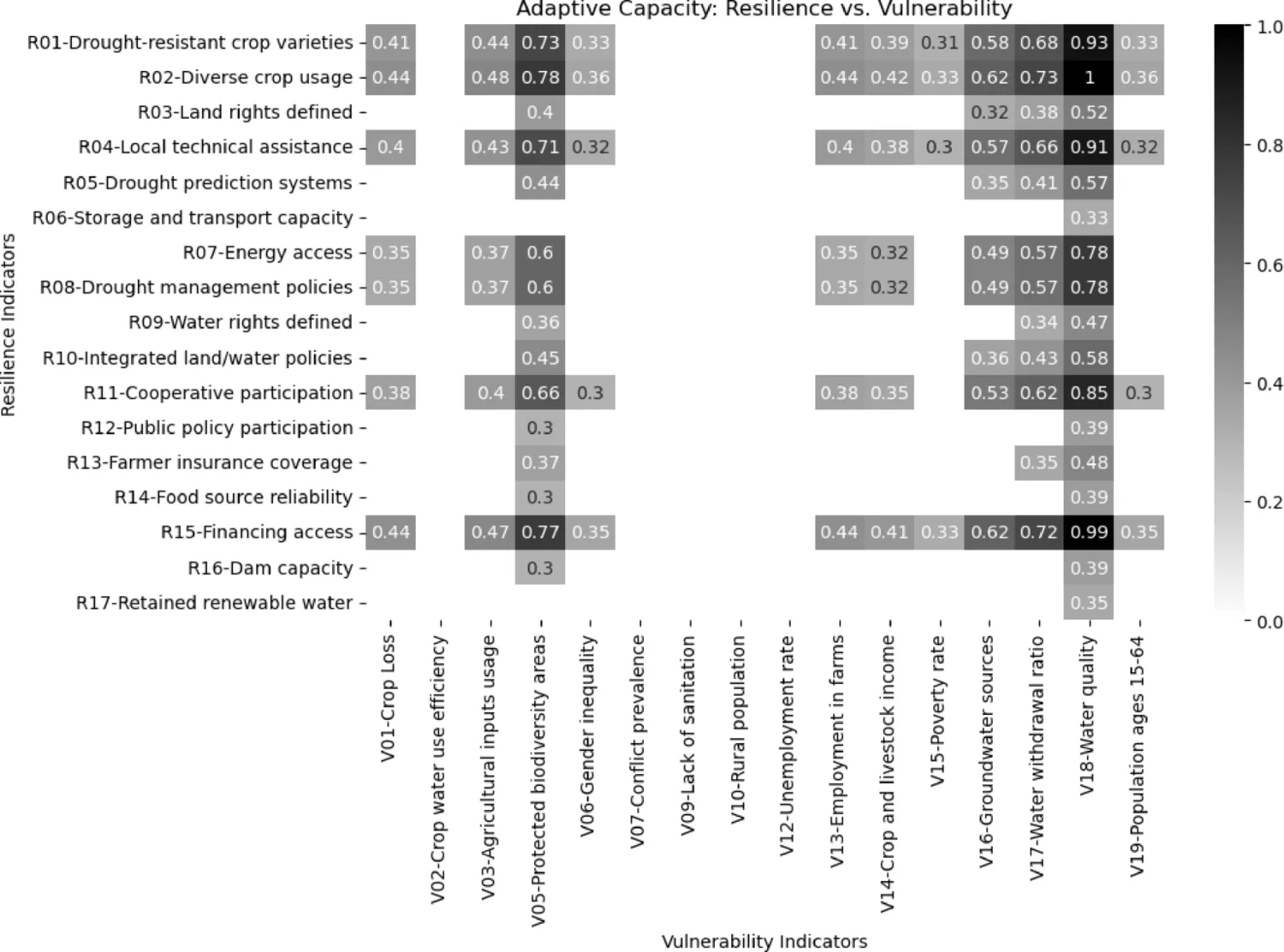
The average normalized ratings for the relationships between the resilience and vulnerability indicators at the adaptive capacity dimension (values < 0.3 were omitted for the clarity of the presentation)

**Fig. 11:**
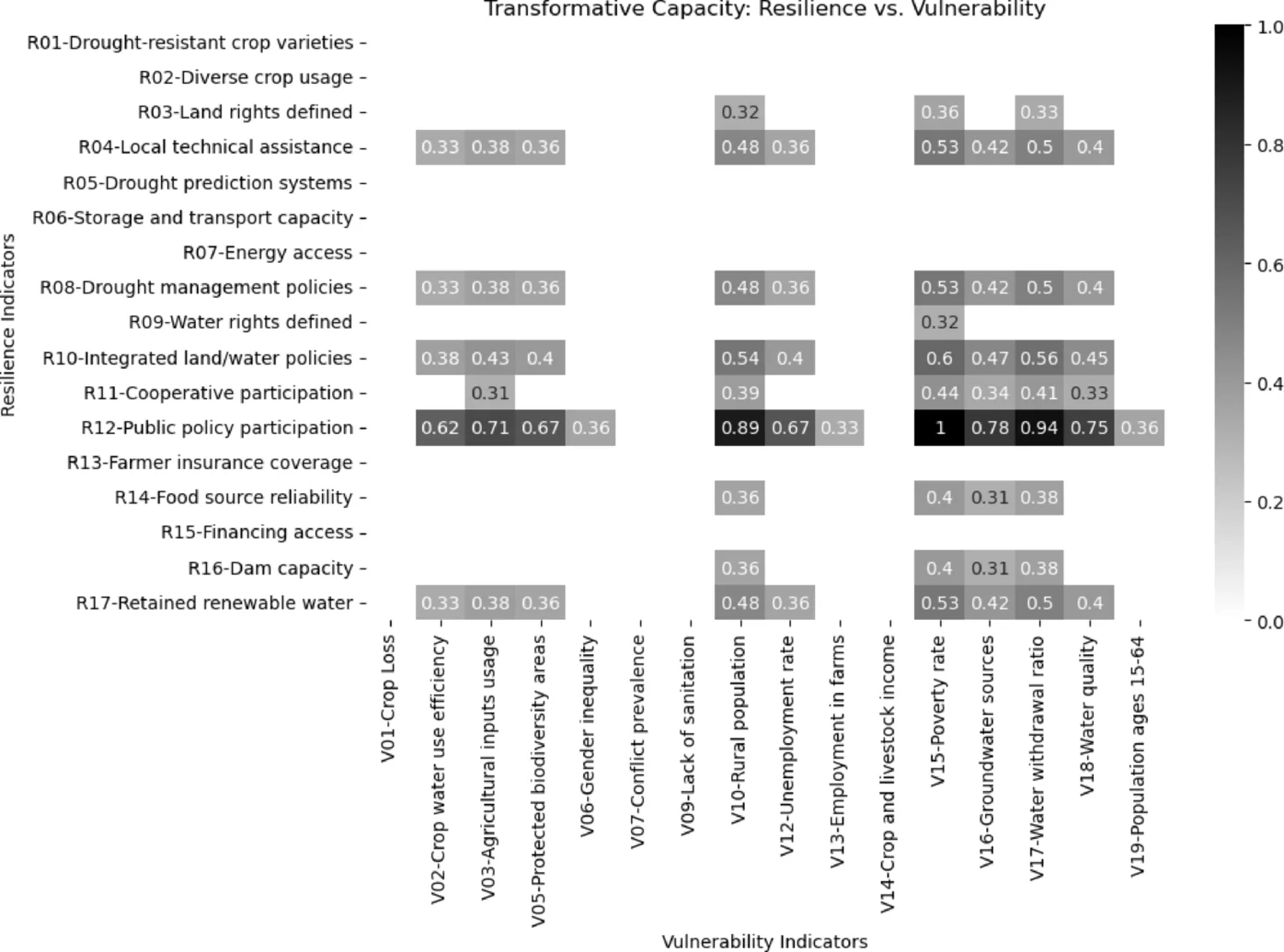
The average normalized ratings for the relationships between the resilience and vulnerability indicators at the transformative capacity dimension (values < 0.3 were omitted for presentation clarity)

This discretion of the temporal scale is subjective, which is a limitation of this case study. However, this approach reduces the number of independent variables that experts have to determine (lower degrees of freedom and less cognitive load). If either component is near zero, the factorization ensures that the combined effective score is near zero, unlike additive forms, which can yield a misleadingly high linkage score when one side is high. Additionally, it helped us to capture a more holistic view of the indicator relationships. Figures 9, 10 and 11 illustrate the calculated $\:{\beta\:}^{s}$ matrix for *s*={Coping, Adaptive, Transformative} capacities. Some observed patterns in the relationships between the resilience and vulnerability indicators are as follows. In the coping dimension, vulnerability indicators V01-Crop Loss, V09-Lack of Sanitation, and V16-Groundwater Sources appear to be particularly important for policymakers to focus on. R04-Local Technical Assistance and R05-Drought Prediction Systems were found to be linked with multiple vulnerabilities, such as V01-Crop Loss, V09-Lack of Sanitation, and V10-Rural Population. Undoubtedly, financial tools, such as R13-Farmer Insurance, are effective in providing relief for farmers due to immediate observable effects due to vulnerabilities in these indicators, such as V01-Crop Loss. However, this resilience indicator is less effective in adapting and transforming capacities to reduce medium- and long-term impact. Like in Study I, the matrix for the adaptive capacity dimension is denser, showing broader coverage across more vulnerabilities. Indicators such as V05-Protected Biodiversity Areas, V17-Water Withdrawal Ratio, and V18-Water Quality are now linked with multiple resilience indicators, suggesting an expanded role in adaptation. Resilience indicators R01-Drought-resistant Crop Varieties, R02-Diverse Crop Usage, and R04-Local Technical Assistance are prominent and have linkages across multiple vulnerabilities. These indicators have a consistently high impact on adaptive capacity. Indicators related to socioeconomic conditions and policy also have more linkages in this dimension compared to the coping dimension.

The transformative capacity matrix (Fig. 11) features limited coverage of vulnerabilities (similar to Study I). Multiple resilience indicators cover vulnerabilities V10-Rural Population, V15-Poverty Rate, and V16-Groundwater Sources, suggesting that transformative capacity is actively targeted at reducing socio-economic disparities and improving water resource management. Policy indicators such as R10-Integrated land/water policies, R11-Cooperative Participation, and R12-Public Policy Participation have more linkages now compared to the other two dimensions, indicating the role that policies play in transformative capacities. Infrastructure-related resilience indicators such as R16-Total Dam Capacity and R14-Food Source Reliability are essential in reducing the structural vulnerabilities of communities facing recurring drought risks in the long term. They help communities build the necessary infrastructure to adapt to changing climatic conditions.

Although we approximated $\:{\beta\:}^{s}$ rather than directly collected data from experts, we observed similar patterns to those in Study I, which examined the connections between indicators. For instance, V01-Crop Loss was identified as a key vulnerability indicator, and the relationship between R13-Farmer Insurance and this indicator was significant in the short term. We also noted a strong connection between R16-Dam Capacity and V16-Groundwater sources at the transformative capacity.

There are also differences between Study I and II. For instance, the total R16-Dam Capacity was mostly considered transformative capacity in Pennsylvania, while the experts regard it as coping capacity in Study I. These differing perspectives stem from the fact that dams in Pennsylvania are primarily used for water management rather than agricultural irrigation. Therefore, the R16-Total Dam Capacity does not represent a short-term coping capacity but rather a long-term water supply. These differences highlight the significance of local knowledge and contextual information in drought risk assessment using indicator-based approaches. Additionally, they underscore the value of examining resilience from various dimensions, as proposed in this framework.

#### Study II: Considering vulnerability and resilience relationships on resilience

**Fig. 12:**
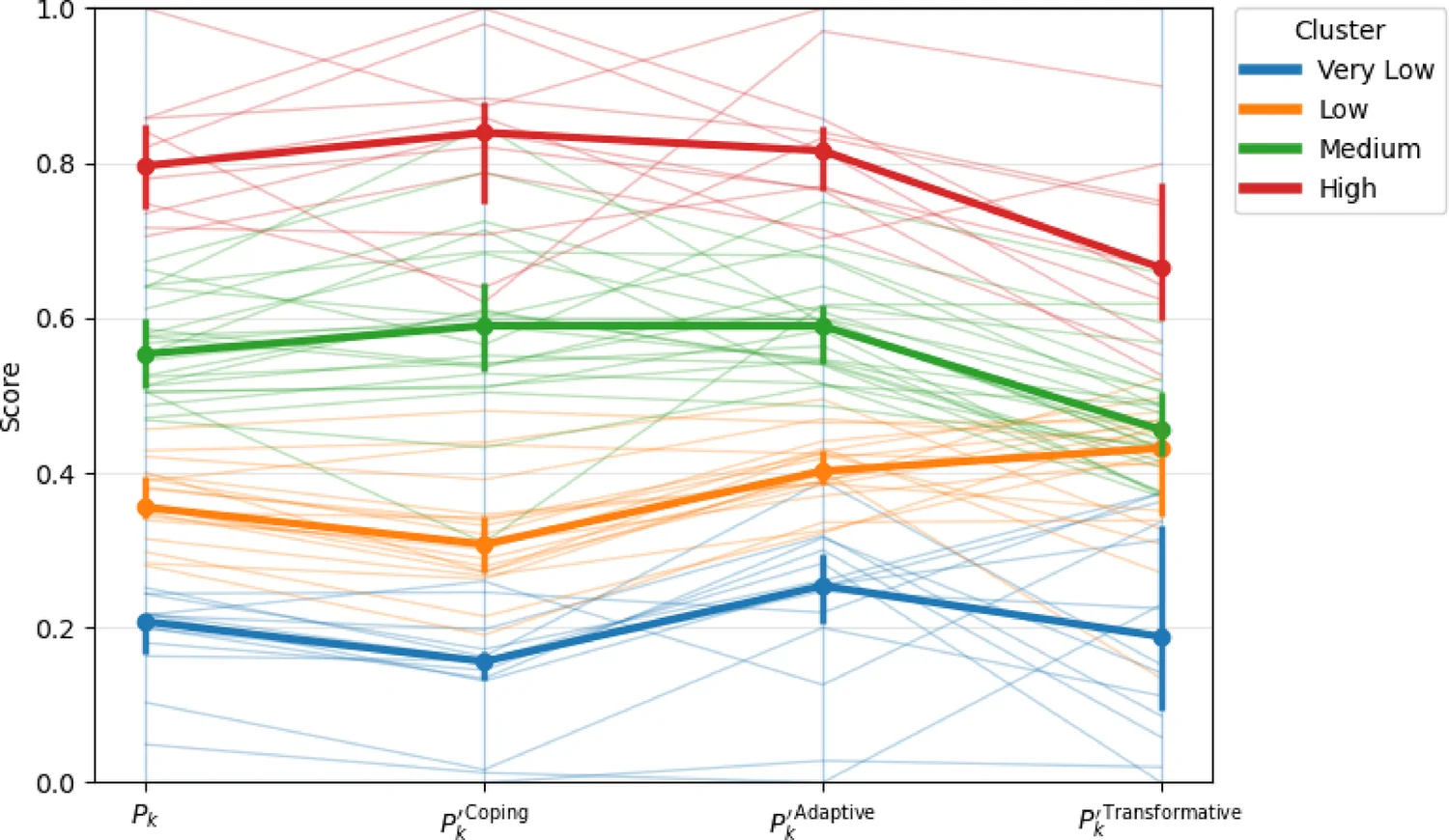
Parallel-coordinates plot of county resilience profiles for the four-cluster solution (Very Low, Low, Medium, High). The first axis shows overall resilience ($\:{P}_{k}$), followed by MADIS capacity-specific scores ($\:{P}_{k}^{{\prime\:}{\hspace{0.17em}}s})$. Thin lines represent individual counties within each cluster. Thick lines show the cluster median profile across dimensions, and vertical tick marks indicate within-cluster interquartile range at each axis

Next, we compared the typical way of measuring the overall resilience, which is calculated as the weighted average of the indicators (i.e., ($\:{P}_{k}$)) and with the proposed MADIS approach (i.e., $\:{P}_{k}^{{\prime\:}{\hspace{0.17em}}s}$). To demonstrate the differences between the two approaches, we used a *K*-means algorithm to cluster the Pennsylvania counties into low, medium, and high resilience based solely on $\:{P}_{k}$. A six-cluster solution produced the highest silhouette score (0.58). However, because our primary objective is to demonstrate the use of MADIS in policy communication and actionable prioritization, we opted for a four-cluster grouping (Very Low, Low, High, Very High with a silhouette score of 0.56) in Fig. 12, which provides more interpretability for decision-making.

Figure 12 presents a parallel coordinates plot of overall resilience $\:{P}_{k}$ and context-dependent resilience $\:{P}_{k}^{{\prime\:}{\hspace{0.17em}}s}$ of the four clusters. As expected, the tiers are clearly ordered by overall resilience $\:{P}_{k}$, which is the base measure used for clustering. The value of MADIS becomes evident in the subsequent axes, where the capacity-specific scores reveal non-parallel trajectories across coping, adaptive, and transformative capacity. In adaptive and transformative capacity, the clear separation among the clusters breaks down, meaning that many counties with high coping capacity may have lower adaptive or transformative capacity. The Low- and Medium-level clusters merge at the transformative capacity level. The High-level cluster shows strong coping and adaptive medians but declines in transformative capacity. This indicates a potential “transformative gap” even among the highest-resilience counties. This observation is not a result of the fact that resilience indicators had higher coping capacity weights (i.e., the coping capacity and overall capacity were correlated); instead, it was because the resilience indicators that are expected to counterbalance vulnerabilities in the transformative dimension had low values (note that the term $\:{P}_{jk}^{s}$ considers only resilience indicators).

This example illustrates why MADIS is more policy-informative than a weighted-average resilience score alone. Therefore, we can state that the MADIS framework offers a more detailed understanding of resilience than simply calculating a weighted average of resilience indicators. By first identifying the types of vulnerabilities present in a system and then evaluating the effectiveness of various resilience mechanisms against these specific vulnerabilities, the proposed framework can provide more comprehensive and actionable insights into overall system resilience. The MADIS framework enables targeted analysis of how different capacities (coping, adaptive, and transformative) address specific vulnerabilities. Consequently, policymakers can use this detailed breakdown to inform decisions on capacity-improvement strategies tailored to the unique needs of different regions. For instance, some areas benefit more from enhanced coping capacities, while regions facing long-term structural challenges may require a focus on transformative capacities. This granular insight may lead to more efficient resource allocation and the development of customized resilience-building strategies, ultimately leading to more effective drought management and climate adaptation policies.

**Fig. 13:**
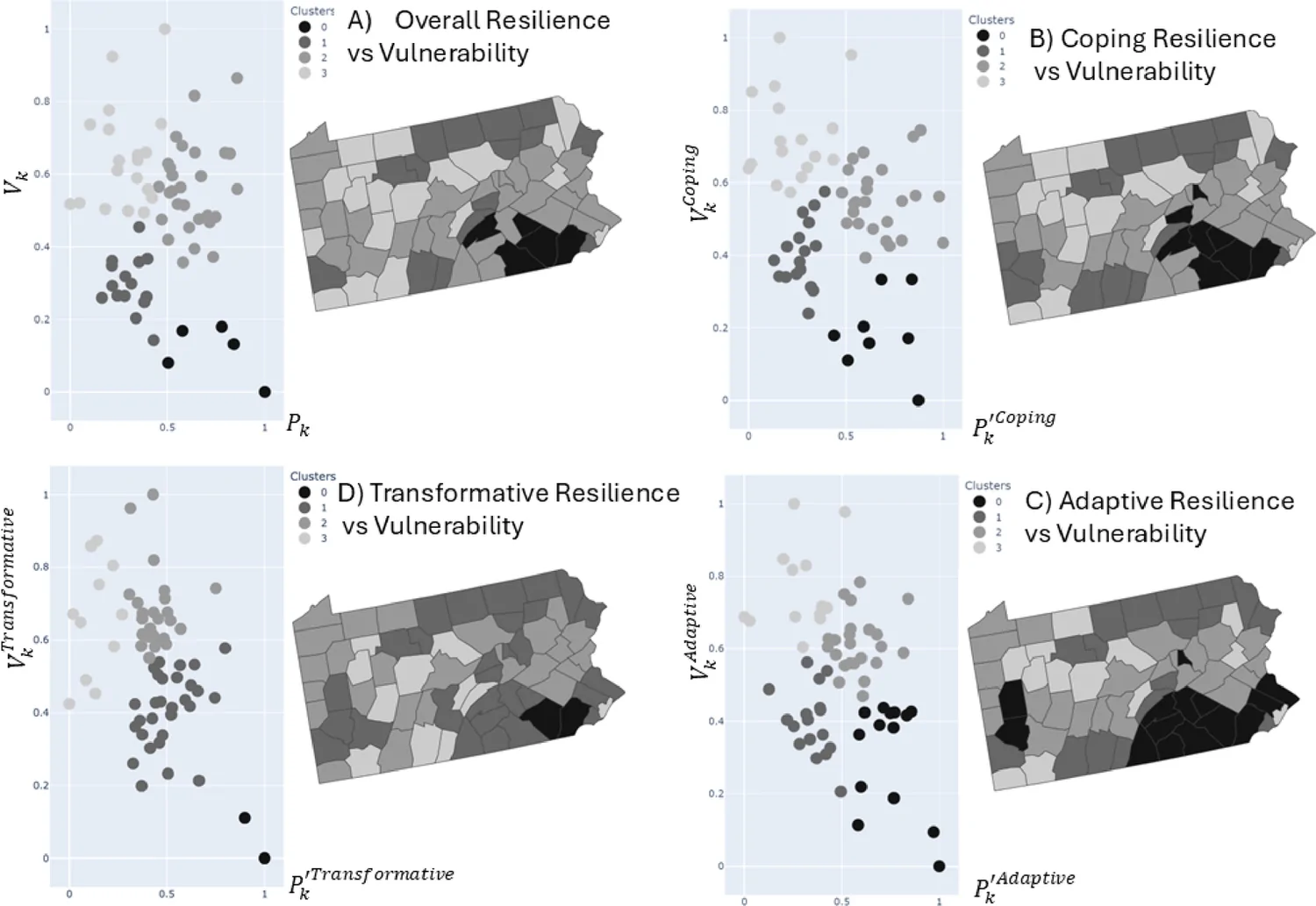
Cluster-based comparison of county typologies under traditional versus MADIS-linked vulnerability–resilience metrics in Pennsylvania. Scatterplots show four-cluster solutions using paired resilience–vulnerability dimensions

To demonstrate how establishing linkages at the indicator level can provide a more nuanced analysis of vulnerability and resilience, we compared $\:{P}_{k}$ and $\:{V}_{k}$ to $\:{P}_{k}^{{\prime\:}{\hspace{0.17em}}s}$ and $\:{V}_{k}^{s}$ using a clustering approach that transforms complex data into actionable insights for guiding drought policy. Figure 13 illustrates the results of the cluster analysis using different resilience and vulnerability metrics as the cluster dimensions. Overall, four clusters emerged for each case. Cluster 0 represents counties with high resilience and low vulnerability; Cluster 1, with low resilience and low vulnerability; Cluster 2, with high resilience and vulnerability; and finally, Cluster 3, with low resilience and high vulnerability. With respect to risk mitigation interventions, the counties in Cluster 3 are the most urgent to prioritize. Clusters 1 and 2 also require different approaches to reduce their drought vulnerability. When we performed the analysis using $\:{P}_{k}^{{\prime\:}{\hspace{0.17em}}s}$ and $\:{V}_{k}^{s}$ where *s*={Coping, Adaptive, Transformative}, some of the counties changed their cluster membership as shown in Fig. 13, indicating their need for different risk mitigation strategies at different dimensions of resilience and vulnerability.

Next, we show how MADIS can be incorporated into an IPCC-based framework for comparison purposes. Hazard and exposure were operationalized at the county level to construct a hazard–exposure index for a multi-objective analysis. Exposure was represented by the number of agricultural producers (* NProducers*) in 2022 (USDA NASS 2022), which was rescaled to [0,1] using min–max normalization. Drought hazard was characterized for each county using two indicators: the Standardized Precipitation–Evapotranspiration Index (SPEI) and the Normalized Difference Vegetation Index (NDVI). Because lower values of SPEI and NDVI indicate drier conditions, both variables were sign-reversed so that larger values correspond to greater hazard, and then min–max normalized to obtain $\:SPE{I}_{k}$ and $\:NDV{I}_{k}$. Then, a composite hazard score was computed as the mean of these standardized indicators. Finally, the hazard–exposure sub index was defined as:

$$\:{HE}_{k}=NProducer{s}_{k}\times\:(SPE{I}_{k}+NDV{I}_{k})/2$$

We constructed a baseline drought-risk index and three capacity-specific risk proxies to compare composite risk estimation with MADIS. The traditional index combines the hazard–exposure pressure term ($\:H{E}_{k}$) with overall vulnerability ($\:{V}_{k}$) and a resilience reduction factor $\left(1-{{P}_{k}}\right)$ as

$$\:{R}_{k}=H{E}_{k}\times\:{V}_{k}\times\:(1-{P}_{k})$$

The above equation states the common assumption that risk increases with hazard/exposure and vulnerability, and decreases as overall resilience increases, and reflects equations given in (Kc et al. 2021; González-Tánago et al. 2016). In the MADIS approach, $\:{V}_{k}^{s}$ denotes the vulnerability score aligned with capacity * s* and is already adjusted for resilience at capacity dimension*s*; hence, a risk index can be computed as follows:

$$\:{MR}_{k}^{s}=H{E}_{k}\times\:{V}_{k}^{s}$$

**Fig. 14:**
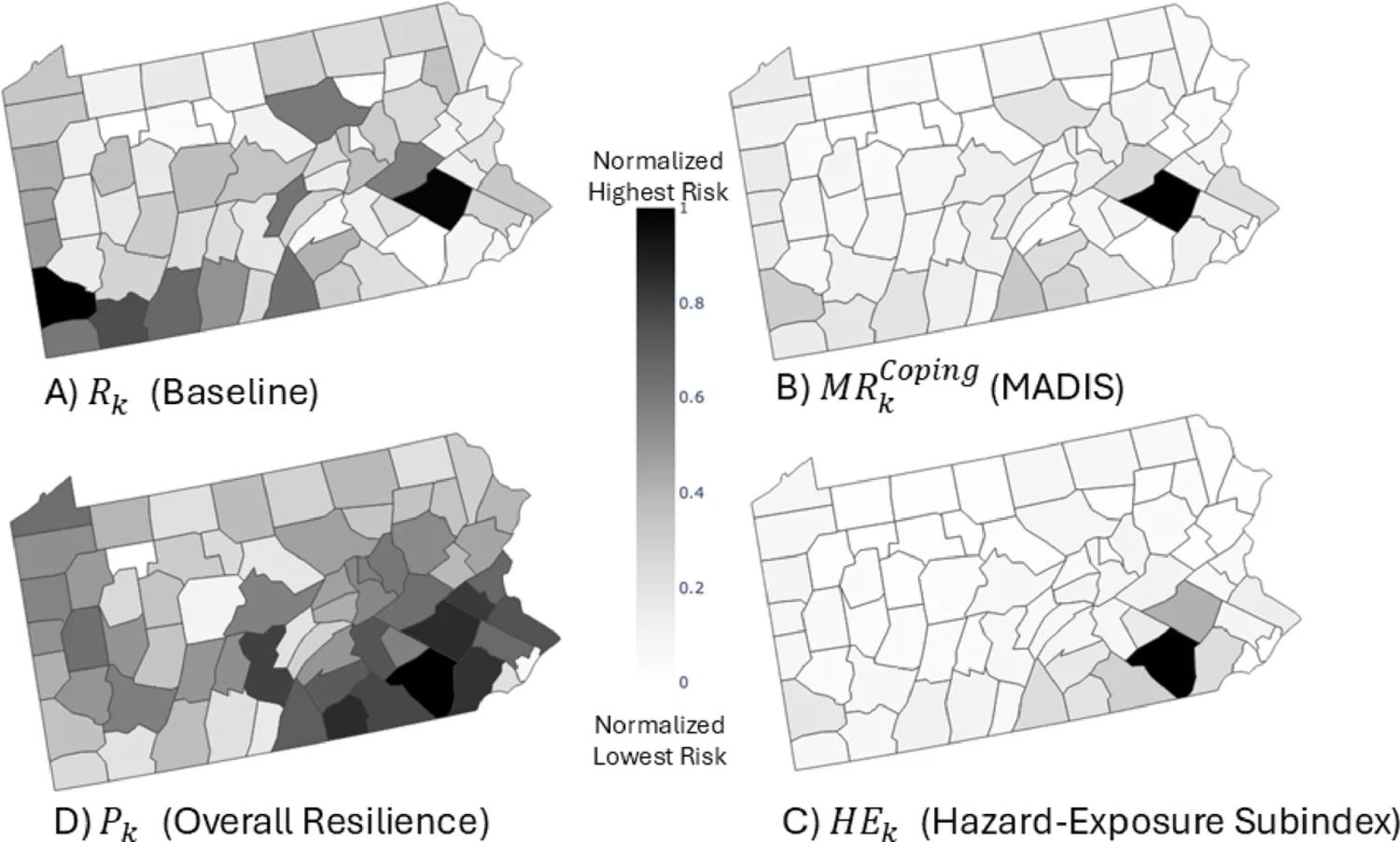
County-level risk patterns under traditional versus MADIS-based assessments in Pennsylvania. The maps compare the spatial distribution of normalized in [0,1] risk derived from a traditional multiplicative

To enable cross-county comparison, the resulting index scores were rescaled to [0,1] using min–max normalization and plotted in Fig. 14. In contrast to $\:{MR}_{k}^{s}$, $\:{R}_{k}$ represents an indicator-based risk formula where vulnerability and resilience indicators are combined after they are aggregated independently.

In Fig. 14, the hazard-exposure hotspot in southeastern Pennsylvania overlaps with a major agricultural belt, including counties with many relatively small farms. The figure only displays $\:{MR}_{k}^{Coping}$ for comparison as the other dimensions exhibit a similar pattern. For example, Lancaster County, one of the state’s most farm-intensive counties, has 4,680 farms with an average farm size of 81 acres; moreover, a substantial share of farms is under 50 acres (USDA NASS, 2022). County rankings based on $\:{R}_{k}$ and $\:{MR}_{k}^{s}$ overlap among top-decile counties. The main difference between the two maps is that the baseline index highlights several counties as moderate-to-high-stress, whereas the MADIS-based map more clearly separates the most-stressed county from the rest. This difference is due to how the indices are constructed. The baseline risk score is an aggregate measure, so different combinations of exposure, vulnerability, and resilience can yield similar composite values, which tends to compress differences among counties into a broad “moderate-to-high” band. In contrast, the MADIS approach relies on indicator-level linkages operationalized in $\:{\boldsymbol{\beta\:}}^{\boldsymbol{s}}$. As a result, a county can have a very high-risk score only when it exhibits specific vulnerabilities and the appropriate combination of linked resilience-vulnerability relationships. Such an alignment will produce a sharper separation in the spatial pattern, which is more useful for policy decisions.

#### Summary of the discussions in study II

Much of the discussion in Study II focused on the operationalizations of the indicators. Therefore, the details of such discussions are beyond the scope of this paper. However, a few distinct views relevant to the scope of this study emerged; some participants questioned the use of broad socioeconomic indicators and suggested more specific indicators for the agricultural sector, while others appreciated the inclusivity of such indicators. While some indicators had clear consensus on their relevance and type, many participants provided varied responses due to differing understandings of the objective, the justification for using the indicator, and what the indicator measured. Some indicators that are very relevant in developing nations, such as population displacement and conflict, were considered unrelated in the context of Pennsylvania. These discussions highlighted that participants from different sectors emphasized indicators specific to their sectors, underscoring the need for collaborative, transdisciplinary approaches to drought resilience. One of the challenges we encountered in adjusting our approach to managing drought in Pennsylvania is that drought policies are primarily designed for non-agricultural water uses, as farming in the state relies primarily on rainfed systems. Respondents suggested that rather than focusing on contextual factors, it might be more suitable to adopt an outcome-driven approach.

## Discussions

### Implications of the proposed MADIS framework and findings

We proposed a novel framework to operationalize the interdependence between resilience and vulnerability by quantifying the effectiveness of resilience indicators in mitigating the potential harm from specific vulnerability indicators under different capacity dimensions (coping, adaptive, and transformative). This section summarizes the practical and theoretical implications of the proposed framework and the case studies in light of the research questions.

R1) *How can the interdependence between vulnerability and resilience be represented in indicator-based frameworks for drought risk analysis?*

This research used a matrix-based structure to represent the interdependencies across three dimensions visually and quantitatively. Furthermore, we demonstrated a process for constructing such a matrix through stakeholder participation to identify which resilience strategies are most effective for specific vulnerabilities. This approach is the main contribution of the research. Participatory and collaborative methodologies involving diverse stakeholders are shown to be a viable approach to modeling the linkages between vulnerability and resilience. In both studies, which were conducted in different modalities and used slightly different methods to collect data, we observed similar patterns regarding the linkages. The collaborative approach was not only helpful in discovering linkages but also facilitated consensus-building among stakeholders. Such engagement can increase the likelihood of successful implementation of the recommended resilience strategies by fostering a shared understanding and support for the proposed interventions. Although the research focuses on drought risk, the same framework and metrics can be adapted to other types of natural hazards, such as floods, wildfires, and hurricanes, where the interaction between vulnerability and resilience is complex and context-specific, in further research.

R2) *How does the effectiveness of resilience and vulnerability indicators in assessing and mitigating drought risk vary across different resilience dimensions?*

Two case studies demonstrated that resilience indicators do not have uniform effectiveness across the coping, adaptive, and transformative capacity dimensions. For example, indicators like “Availability of drought prediction and warning systems” and “Technical assistance from local entities” were seen as critical in the short term to prevent immediate crop losses and water shortages. These indicators were effective for rapid response and stability but limited in terms of transformative capacity effectiveness. Transformative indicators that measured structural transformation and long-term resilience building included “Integrated land and water management policies,” “Public policy participation,” and “Existence of drought management policies.” Such indicators were associated with governance and policy-related vulnerabilities. A practical implication of this differentiation is to help policymakers and practitioners identify which specific resilience indicators should be prioritized depending on the desired outcome (e.g., immediate relief versus long-term systemic change). This differentiation within our MADIS framework also allows a more detailed scenario and policy trade-off analysis by measuring changes in risk levels when specific indicators are strengthened or weakened. The framework can be directly incorporated into a decision support system (DSS) to provide multi-dimensional drought risk profiles for regions or communities. For example, the DSS can show that a region has a high coping capacity but a low transformative capacity. Such an analysis can guide strategic investments.

This research has demonstrated that the relationship between drought vulnerability and resilience is complex and context-dependent. This observation has several theoretical implications for further research. Firstly, it challenges the oversimplified view of existing theoretical frameworks that analyze resilience and vulnerability at the aggregate level. Further research is needed at the indicator-specific level to capture the dynamics between resilience and vulnerability. Such research should focus on quantitative analysis of indicator-level relationships and methodological approaches to better capture these dynamics. Secondly, the research supports the view that resilience should be understood as a dynamic process rather than a static state influenced by multiple interacting factors that evolve. The frameworks summarized in Sect.  2.2 also support this notation. To fully understand drought resilience as a dynamic process, further longitudinal studies are needed to track the progression of coping, adaptive, and transformative capacities over time.

R3) *To what extent does considering the interdependencies between vulnerability and resilience indicators at different dimensions enhance the predictive accuracy of indicator-based drought risk models?*

The results showed that the inclusion of interdependencies can lead to more precise risk categorizations. In Study II, integrating the interdependencies led to more distinct separations between high, medium, and low-risk counties, suggesting that the proposed framework better captured the complexities of drought risk. Our framework requires more involved, diverse stakeholder input to investigate interdependencies compared to aggregate-level analysis, where stakeholder input is used to identify indicator weights. The participants found it challenging to investigate the relationships between agricultural, social, technical, and environmental indicators. However, their feedback confirmed that modeling these interdependencies is more aligned with the real world and can lead to a better understanding of drought resilience, ultimately improving policymaking. This can be achieved using the social-technical-ecological systems (SETS) approach. Further research should explore how the SETS approach can be integrated into the process-based view of resilience. Specifically, further research should analyze how different components of the SETS framework influence the transition between coping, adaptive, and transformative capacities. Further research is also needed to better understand how incorporating interdependencies can offer more actionable insights for decision-makers and policymakers.

### Limitations of the research and further directions

One of the primary limitations of the research is the context-specific nature of the selected indicators for vulnerability and resilience. The indicators and interdependencies defined in the research may not be universally applicable across different regions or types of droughts. The framework relies on the specific vulnerabilities and resilience strategies relevant to the case studies (e.g., Pennsylvania and small-scale farming communities), which might limit its generalizability to other socio-ecological contexts, such as urban drought resilience or large-scale farming. It should be re-emphasized that this paper focuses on presenting a new framework for indicator-based drought resilience and vulnerability assessment, rather than addressing drought risk in a specific area. The present approach is limited to static cross-sectional studies, which capture conditions at a single time point and do not represent temporal dynamics or changing hazard regimes. Since Study I and II used small, non-comparable samples, their differences are not generalizable and require validation with larger, more diverse groups.

One limitation of the framework is that it treats temporal scales as discrete categories. Future iterations could incorporate more continuous temporal effects or feedback loops between different time scales. Further research will also consider incorporating hazard indicators into MADIS. This requires an analysis of the hazard-to-vulnerability mapping. Similar to vulnerability-to-resilience mapping presented in this paper, further research can quantify how hazard characteristics interact with vulnerabilities associated with coping, adaptive, and transformative dimensions, and evaluate how resilience mechanisms counterbalance these hazard-sensitive vulnerabilities.

The MADIS-based risk assessment in 5.5.2 is preliminary, demonstrating how to operationalize indicator-level linkages between vulnerability and resilience to inform policy. The main focus of the two studies is on the linkages between resilience and vulnerability. Future work can extend MADIS by explicitly modeling hazard–vulnerability relationships alongside these linkages, enabling a more complete representation of how exposure, susceptibility, and capacity interact across contexts.

## Conclusions

While vulnerability and resilience are typically analyzed at an aggregate level, the relationships between individual indicators remain underexplored. This paper introduces a new analytical framework that quantifies the effectiveness of resilience indicators in mitigating specific vulnerabilities. This granular approach allows for more targeted risk assessments and policy interventions, providing clearer guidance for enhancing resilience and reducing vulnerability at the individual indicator level.

## Appendix

See Tables 4, 5 and 6.

**Table 4:** Description of the resilience indicators used in study II (*Note: All resilience indicators were min-max normalized to a [0, 1] scale where 1 represents the highest level of resilience in the calculations given in the paper*.)

No	Indicator	Short name	Calculation/definition	Source of data
R01	Percentage of drought-resistant crop varieties cultivated	R01-Drought-resistant crop varieties	Indicator initially deemed not applicable for Pennsylvania context	
R02	Percentage of farmers who use different types of crops	R02-Diverse crop usage	We calculated the Simpson Index of Diversity (SID) for this indicator using the area planted/used for each crop and livestock. The total area harvested for crops was used for all crops listed by the National Agricultural Statistics Service (NASS) for each county. Standard calculation from SID equation: $\:D=\:{\sum\:}_{i=1}^{R}\left(\frac{{n}_{i}({n}_{i}\:-\:1)}{N(N\:-\:1)}\right)$ where n_i_ is the number of areas harvested for species “i” in the county, and N is the total number of harvested areas in the county	USDA National Agricultural Statistics Service (2017). NASS - Quick Stats. USDA National Agricultural Statistics Service
R03	Land rights clearly defined (yes/no)	R03-Land rights defined	Yes, for all of Pennsylvania	
R04	Technical assistance from local entities (e.g., cooperatives/NGO/government)	R04-Local technical assistance	Yes, to all PA, county extensions are available to all	
R05	Availability of drought prediction and warning systems or climatic predictions	R05-Drought prediction systems	Yes, to all of Pennsylvania, US Drought Monitor is available for all counties	
R06	Produce storage and transportation capacity	R06-Storage and transport capacity	The total grain storage capacity for each county is expressed in BU	USDA National Agricultural Statistics Service (2017). NASS - Quick Stats. USDA National Agricultural Statistics Service
R07	Access to energy	R07-Energy access	We used the percent of household income used towards energy cost (energy burden) for this indicator. This information is available for the United States through the Department of Energy. This value was inverted (1-value) as the high energy burden is negative	Ma, Ookie, Krystal Laymon, Megan Day, Ricardo Oliveira, Jon Weers, and Aaron Vimont. 2019. Low-Income Energy Affordability Data (LEAD) Tool Methodology. Golden, CO: National Renewable Energy Laboratory. NREL/TP-6A20-74249
R08	Existence of drought management policies (mitigation/adaptation/prevention/preparedness)	R08-Drought management policies	Yes, to all of Pennsylvania. It is required by law for counties to have drought management policies. Pennsylvania Code Title 4 Chap. 118 requires each public water supplier in a drought emergency area to develop a drought contingency plan or update an existing one if necessary and submit it to the Commonwealth Drought Coordinator for review and approval	
R09	Water use rights clearly defined	R09-Water rights defined	Yes to all of Pennsylvania, Water rights are clearly defined for all of PA	
R10	Integrated land and water management policies	R10-Integrated land/water policies	Indicator initially deemed not applicable for Pennsylvania context	
R11	Participation in farming cooperatives or associations	R11-Cooperative participation	The total number of farm co-ops and CSAs within the county were summed	KDC Cooperative Solutions. Map of Cooperatives. CRAFTchatham Regional Food Systems Inventory PA-OH-WV. 2022. Producers PA-OH-WV. Accessed: 12.03.2023
R12	Level of public participation in local policy	R12-Public policy participation	Indicator initially deemed not applicable for Pennsylvania context	
R13	Percentage of farmers with crop, livestock, or drought insurance	R13-Farmer insurance coverage	Total number of farmers/farms with insurance is unknown. Instead, the total number of policies taken in each county for each year was used and divided by the total number of operations in the county	USDA National Agricultural Statistics Service (2017). NASS - Quick Stats. USDA National Agricultural Statistics Service. USDA: Risk Management Agency (2018–2022), State/County/Crop Summary of Business. Accessed: 12/01/23
R14	Food source reliability and diversity	R14-Food source reliability	It is calculated by dividing the area considered to be a food desert by the total county area. A food desert area is defined in our calculation as areas where there are low-income and low-access areas more than a half mile away from the nearest supermarket in urban areas or farther than 10 miles in rural areas. Low-income rural areas where a significant number of households do not have cars are also considered food deserts. The normalized value is inverted by 1-value as the percent food desert is a negative trait	USDA Economic Research Service. 2019. Food Access Research Atlas (FARA)
R15	Access to financing and credit	R15-Financing access	Yes for all of Pennsylvania	
R16	Total dam capacity	R16-Dam capacity	The total dam capacity in Acre-Ft is summed for each county	National inventory of dams. Washington, DC: US Army Corps of Engineers: Federal Emergency Management Agency
R17	Percentage of retained renewable water	R17-Retained renewable water	Indicator deemed not applicable for Pennsylvania context. This refers to collected rainwater and recycled water. A practice that is important in arid climates	

**Table 5:** Description of the vulnerability indicators used in study II (*Note: All vulnerability indicators were min-max normalized to a [0, 1] scale where 1 represents the highest level of vulnerability in the calculations given in the paper.*)

No	Indicator	Short name	Calculation/definition	Source of data
V01	Crop Damage & Sensitivity (Crop Loss)	V01-Crop Loss	This indicator was calculated in relation to insurance data through USDA Risk Management Agency (RMA). Crop loss was calculated by dividing the total number of insurance units indemnified for crop loss by the total number of crop units covered. This is a calculated average from the previous 5 years. This value was inverted (1-value) as high crop loss is a negative trait	USDA: Risk Management Agency (2018–2022), State/County/Crop Summary of Business. Accessed: 12/01/23
V02	Crop water use efficiency (WUE)	V02-Crop water use efficiency	Indicator deemed not applicable for Pennsylvania context.	
V03	Use of insecticides and pesticides (Use of agricultural inputs)	V03-Agricultural inputs usage	Calculated by summing mass of all pesticides used over one year in each county. Pesticide use is positive in this case as it shows farmers can afford pesticide usage and farms will be less sensitive to drought	Wieben, C.M., 2019, Estimated Annual Agricultural Pesticide Use for Counties of the Conterminous United States, 2013-17 (ver. 2.0, May 2020): U.S. Geological Survey data release
V04	Degree of land degradation and desertification	V04-Land degradation	Indicator initially deemed not applicable for Pennsylvania context	
V05	Percentage of area protected and designated for the conservation of biodiversity.	V05-Protected biodiversity areas	Calculated by adding all conserved area types together in ArcGIS and dividing by the county’s total area to calculate the percent area within each county. Conserved land categories included in the total conserved land area calculation were: Agricultural Security Areas, Conservation Easements, Farmland Preservation Easements, Federal, Local, State, and Privately Owned	WeConservePA. 2023. PA Conserved Land. Accessed through PASDA
V06	Gender inequality	V06-Gender inequality	Percentage of farm managers that are female Source: USDA National Agricultural Statistics Service (2017). NASS - Quick Stats. USDA National Agricultural Statistics Service	
V07	Prevalence of rural conflict/insecurity	V07-Conflict prevalence	This does not apply to the Pennsylvania context	
V08	Percentage of population displaced internally or transboundary	V08-Displaced population	Indicator initially deemed not applicable for Pennsylvania context	
V09	Percentage of population without access to (improved) sanitation	V09-Lack of sanitation	We used percentage area coverage by community water systems (CWS) in the entire county area. This was calculated by finding the area within and outside of CWS using ArcGIS. Although this does not directly affect sanitation, it does affect controlled clean water access	Pennsylvania Department of Environmental Protection. 2023. Public Water Supplier’s (PWS) Service Areas. Accessed through PASDA
V10	Percentage of rural population	V10-Rural population	Percentage of population that is considered rural within each county	U.S. Census Bureau, 2010 Census. Table: P2
V11	The presence of drivers of migration and displacement	V11-Migration drivers	Indicator initially deemed not applicable for Pennsylvania context	
V12	Unemployment rate	V12-Unemployment rate	County-level unemployment rate percentage. This value was inverted by a 1-normalized value	Pennsylvania Department of Labor and Industry. 2023. Local Area Unemployment Statistics.
V13	Percentage of the population employed in small farms	V13-Employment in farms	We used the percentage of the county population employed as a producer for this indicator. Although there is no data on how many are employed on family farms. Family Farm is defined by USDA as “a farm that is closely held by one or more households, generally including the operator’s household. A family farm may be a sole proprietorship, partnership, or family corporation. Farms that are not family farms are farms operated by a hired manager and farms organized as nonfamily corporations or cooperatives.” Area harvested on family farms is available from the Agricultural Statistics Survey	USDA National Agricultural Statistics Service (2017). NASS - Quick Stats. USDA National Agricultural Statistics Service.
V14	Percentage of crop and livestock production in the income of smallholder farms	V14-Crop and livestock income	Relative income from farming for individuals associated with small farms could not be found in census data at the county level. It is also known that most small farms in the US have net losses to total income. We did not use this indicator for Pennsylvania	
V15	Poverty Rate	V15-Poverty rate	The percentage of the county population living below the poverty line averaged over the most recent year. The normalized value is inverted (1-value) as the poverty percentage is a negative trait	U.S. Census Bureau, 2020 Census. Table: S1701
V16	Groundwater level/sources	V16-Groundwater sources	Average depth to water table over the most recent 10-year data period for each county observation well. If data from multiple wells, these values were averaged. After normalization, this value was inverted (1-value) as areas with higher water tables are considered a positive attribute to drought resilience	U.S. Geological Survey, 2016, National Water Information System data available on USGS Water Data for the Nation
V17	Baseline water stress (ratio of withdrawals to renewable supply)	V17-Water withdrawal ratio	County centroids (lat., long.) were used to retrieve baseline water stress data. The normalized value was inverted (1-indicator) because higher water stress is a negative trait	Kuzma, S., M.F.P. Bierkens, S. Lakshman, T. Luo, L. Saccoccia, E. H. Sutanudjaja, and R. Van Beek. 2023. “Aqueduct 4.0: Updated decision-relevant global water risk indicators.” Technical Note. Washington, DC: World Resources Institute
V18	Water quality (categorical)	V18-Water quality	The indicator was initially deemed not applicable to the Pennsylvania context	
V19	Percentage of population ages 15–64	V19-Population ages 15–64	The percentage of the county population between the ages of 15 and 64	Pennsylvania Department of Labor and Industry. 2023. Center for Workforce Information and Analysis

**Table 6:** Description of the hazard and exposure indicators used in study II

Indicator	Definition/calculation	Source
Standardized Precipitation Evapotranspiration Index (SPEI)	This index reports drought severity according to intensity and duration, considering both precipitation and potential evapotranspiration (PET). Value averaged over a 100-year period. We use the Global SPEI database (SPEIbase) to report already calculated SPEI for county centroids (lat., long.)	Santiago Beguería (2022). sbegueria/SPEIbase: Version 2.7 (v2.7). Zenodo. https://doi.org/10.5281/zenodo.5864391
Normalized Difference Vegetation Index (NDVI)	NDVI measures the health and density of vegetation using satellite imagery data using near-infrared and visible light reflectance. We report 20-year NDVI averages for county centroids (lat., long.) using NASA’s current global remotely sensed data reported through MODIS/Terra Vegetative Indices	**Source**: USDA National Agricultural Statistics Service (2017). NASS - Quick Stats. USDA National Agricultural Statistics Service.
